# Deciphering the emergence, genetic diversity and evolution of classical swine fever virus

**DOI:** 10.1038/s41598-017-18196-y

**Published:** 2017-12-20

**Authors:** Liliam Rios, Liani Coronado, Dany Naranjo-Feliciano, Orlando Martínez-Pérez, Carmen L. Perera, Lilian Hernandez-Alvarez, Heidy Díaz de Arce, José I. Núñez, Llilianne Ganges, Lester J. Pérez

**Affiliations:** 1 University of New Brunswick, Saint John, New Brunswick E2L4L5 Canada; 2 Centro Nacional de Sanidad Agropecuaria (CENSA), La Habana, 32700 Cuba; 3 Universidad de las Ciencias Informáticas (UCI), La Habana, Cuba; 4 Hospital Italiano de Buenos Aires, Juan D. Perón 4190, C1181ACH Buenos Aires, Argentina; 5 IRTA-CReSA. Centre de Recerca en Sanitat Animal, Barcelona, 08193 Spain; 6 OIE Reference Laboratory for Classical Swine Fever and OIE Collaborative Centre for Research and Control of Emerging and Re-emerging Swine Diseases in Europe, IRTA-CReSA, Barcelona, Spain; 7 Dalhousie University, Dalhousie Medicine New Brunswick, Saint John, New Brunswick E2L4L5 Canada

## Abstract

Classical swine fever (CSF) is one of the most important infectious diseases causing significant economic losses. Its causal agent, CSF virus (CSFV), is a member of the *Pestivirus* genus included into the *Flaviviridae* family. Previous molecular epidemiology studies have revealed the CSFV diversity is divided into three main genotypes and different subgenotypes. However, the classification system for CSFV has not yet been harmonized internationally. Similarly, the phylogeny and evolutionary dynamics of CSFV remain unclear. The current study provides novel and significant insights into the origin, diversification and evolutionary process of CSFV. In addition, the best phylogenetic marker for CSFV capable of reproducing the same phylogenetic and evolutionary information as the complete viral genome is characterized. Also, a reliable cut-off to accurately classify CSFV at genotype and subgenotype levels is established. Based on the time for the most recent common ancestor (tMRCA) reconstruction and cophylogenetic analysis, it was determined that CSFV emerged around 225 years ago when the Tunisian Sheep Virus jumped from its natural host to swine. CSFV emergence was followed by a genetic expansion in three main lineages, driven by the action of positive selection pressure and functional divergence, as main natural forces.

## Introduction

Classical swine fever (CSF) is a highly contagious viral disease, considered one of the most important infectious diseases that affect domestic pigs and wild boar (*Sus scrofa*)^1^. Because of its huge economic impact, the disease is notifiable to the OIE^2^. CSF was first described in southern Ohio along the Muskingum River in 1833 and in the Wabash River area of Indiana from 1830 to 1833. By the 1860s^3^ the disease was widespread in Europe and America. Currently, CSF has been successfully eradicated from some countries including the United States, Australia and New Zealand; however, it continues to have a severe impact on Asia, Eastern Europe and most of South and Central America as well as the Caribbean^4^. In the European Union (EU), a progressive eradication program was implemented beginning in the early 90′s, which was based on a non-vaccination/stamping out policy and the restriction of movement of animals and their products^5^. Nevertheless, outbreaks keep occurring due to viral introduction via feral pigs (wild boar) or foreign imports and, as expected under the non-vaccination policy, these CSF-outbreaks have caused huge economic losses in areas with a high-density of pigs^1^.

CSF is caused by CSF virus (CSFV), a small-enveloped RNA virus of the genus *Pestivirus* included into the *Flaviviridae* family. The CSFV genome is a single plus-strand RNA, which contains one large open reading frame (ORF) flanked by two untranslated regions (UTRs). The ORF encodes a polyprotein of approximately 3900 amino acids which is subsequently processed by cellular and viral proteases into mature proteins: four structural proteins (C, Erns, E1 and E2) and 8 non-structural proteins (Npro, P7, NS2, NS3, NS4A, NS4B, NS5A, NS5B)^6^. Based on these different genomic regions, studies of molecular epidemiology have been conducted, which have revealed that the diversity of CSFV comprises three main genotypes and different subgenotypes^7^. However, no international consensus of the classification system for CSFV has been reached^4^. Thus, different strains classified as novel subgenotypes without harmonized criterion has been recently reported^8–12^. Likewise, the phylogeny and evolutionary dynamics of CSFV remain unclear. A recent study indicated that CSFV emerged as long ago as 2770.2 years at a rate of 13 × 10^−4^ substitutions per site per year^13^. However, these results were not supported by epidemiological evidence regarding the origin of CSFV or by co-phylogenetic analyses.

Additionally, the evolutionary forces driving the evolution and diversity of CSFV have been poorly studied and are still an open question. Only a few studies using field isolates have addressed this important aspect. Perez *et al*.^14^ showed that the vaccination program implemented as control measure in the Cuban swine herds has led to positive selection on B/C domain of the E2 protein for viral isolates circulating in Cuba (subgenotype 1.4). This event was also linked to a decrease in the virulence of the strains and the viral escape from the host immune response^14–16^. Meanwhile, Ji *et al*.^17^ and Hu *et al*.^18^ both found that vaccination could affect CSFV diversity and lead to the evasion of the immune response through recombination and point mutation, influencing the population dynamics, evolutionary rates and adaptive evolution of CSFV. However, both studies were focused only on viral strains of subgenotypes 1.4 and 2.1 respectively. Therefore, the evolutionary forces causing the viral diversity of other CSFV subgenotypes remain unknown.

To address some of the uncertainty surrounding the origin, genetic variability and evolutionary process of CSFV, we applied phylogenetic inference, homology modelling and phylodynamic and host-virus simulation techniques. In the current study, the reliability of the most commonly used phylogenetic markers for CSFV classification and evolutionary studies was assessed. The evolutionary forces including the positive selection pressure and the adaptation among the different CSFV-lineages were also analyzed. The evolutionary history of CSFV lineages was estimated by time-calibrated phylogenomic approach. To get a better understanding of the relationship between CSFV and the remaining members of *Pestivirus* genus with their respective vertebrate hosts, a co-evolutionary analysis was also performed.

## Results

### Phylogenetic marker assessment

**Figure 1:**
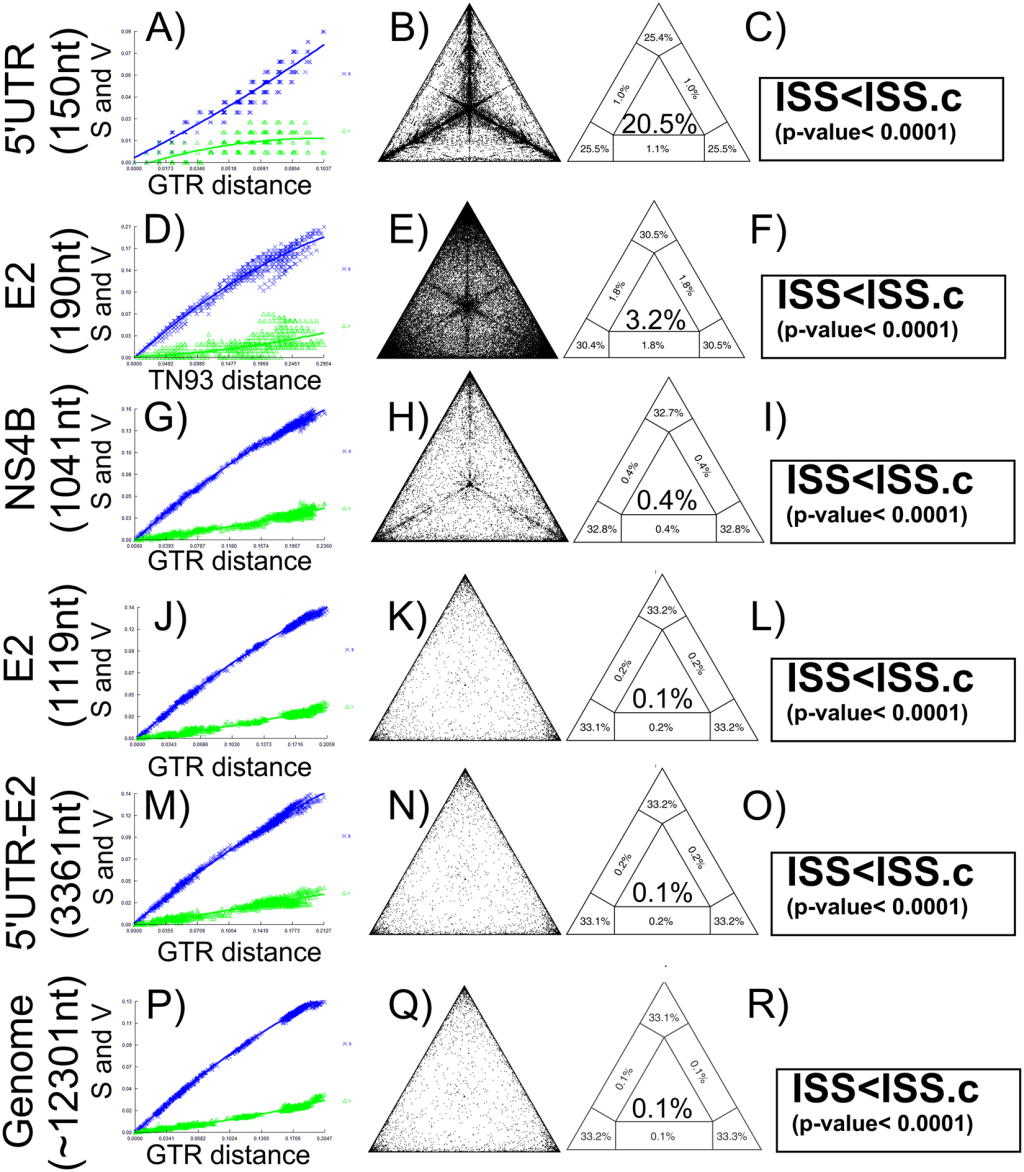
Evaluation of homoplasious signal and phylogenetic noise of different molecular markers for CSFV. The markers proposed for the clasification of CSFV and the complete genome were denoted: (**A**–**C**) 5′UTR^24^, (**D**–**F**) E2 (190 nt)^53^, (**G**–**I**) NS4B^13^, (**J**–**L**) E2 (1119 nt)^52^, (**M**–**O**) 5′UTR-E2^52^, (**P**–**R**) respresent the complete genome used as gold standard. (**A**,**D**,**G**,**J**,**M** and **P**) plot representation of the number of transitions and transversions versus the genetic distance calculated with the best fitted model obtained by jmodeltest^55^; (**B**,**E**,**H**,**K**,**N** and **Q**) likelihood mapping of CSFV sequences, the dots inside the triangles represent the posterior probabilities of the possible unrooted topologies for each quartet. Numbers indicate the percentage of dots in the centre of the triangle corresponding to phylogenetic noise; (**C**,**F**,**I**,**L**,**O** and **R**) results obtained from the Xia’s-test^23^ and the statistical support for each sequence group is also shown.

Saturation effects were investigated by plotting the absolute number of transitions and transversions versus genetic distance for the complete genome of CSFV and for all phylogenetic markers studied (Fig. 1). The number of observed transversions relative to that of transitions gradually increased with growing divergence, for all datasets, except for the phylogenetic marker 5′UTR (150 nt) (Fig. 1A). For this phylogenetic marker, the absolute number of transversions showed a similar pattern to an asymptotic curve, indicating saturation. For the remaining phylogenetic markers and the complete genome of CSFV, the transitions and transversions were not saturated.

The phylogenetic noise in each dataset was investigated using likelihood mapping. The percentage of dots inside the central area of the triangles ranged from 0.1% for the complete CSFV genome and the whole E2 gene (1119 nt), to 20.5% for the 5′-UTR marker (Fig. 1). None of the datasets showed more than 30% noise, which made them suitable to deduce the phylogenetic signal. However, the loss of information was considerably important for the phylogenetic markers 5′-UTR and E2-partial (190 nt), whereas the noise was below 1% for the remaining phylogenetic markers. The phylogenetic marker of the complete E2 gene showed the same loss as the complete viral genome (Fig. 1K and Q). On the other hand, Xia’s test did not support saturation for any of the phylogenetic markers assessed (Iss < Iss.c, p < 0.0001) (Fig. 1).

**Figure 2:**
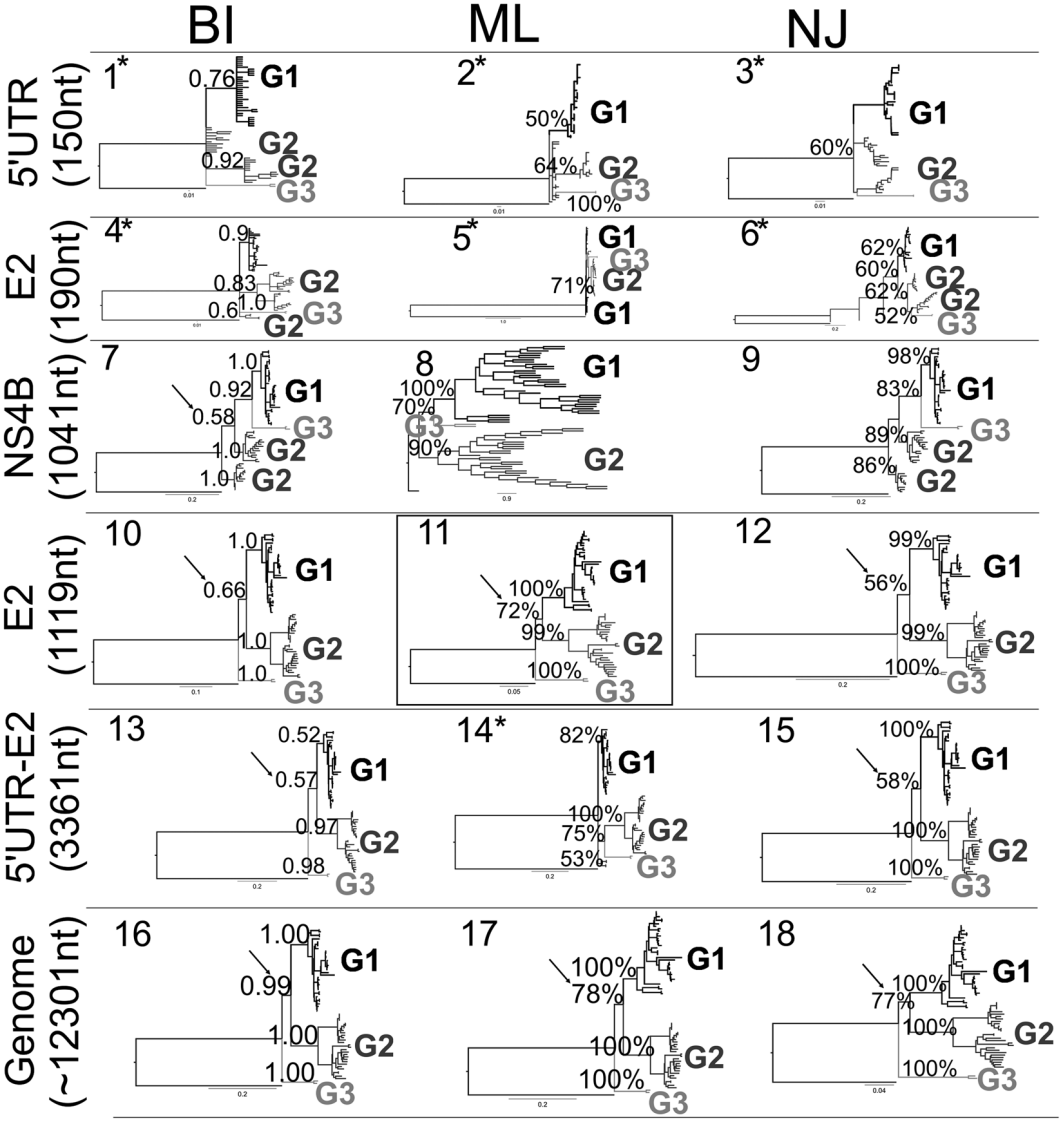
Phylogenetic tree topology comparison. Topologies obtained from the different markers included in the current study (5′UTR^24^, E2 (190 nt)^53^, NS4B^13^, E2 (1119 nt)^52^, 5′UTR-E2^52^) and the complete CSFV genome by Bayesian Inference (BI), maximum likelihood (ML) and Neighbour Joining (NJ) analyzes are shown. The three main linages found for CSFV were denoted (CSFV-genotype 1 (G1), CSFV-genotype 2 (G2) and CSFV-genotype 3 (G3)). The number for each phylogenetic tree corresponds with the number in the topology comparison Table obtained from Kishino and Hasegawa test (K–H)^58^ and the Shimodaira–Hasegawa test (S–H)^59^ (Supplementary Table S1). The best phylogenetic tree estimated by K-H and S-H as well as by statistical support for the internal nodes was bounded by continuous lines. The internal node where the divergence of the CSFV-G2 was statistically supported is denoted with a black arrow. The phylogenetic trees with statistically significant difference with the best selected topology were highlighted by asterisks.

The phylogenetic relationship between the CSFV strains based on complete viral genome and all the different phylogenetic markers were assessed by means of Neighbour Joining (NJ), Bayesian Inference (BI) and Maximum Likelihood (ML). Reconstructions of these relationships are shown in Fig. 2. Different topology structures for the phylogenetic trees were obtained depending on the phylogenetic marker or the analysis used. From all the phylogenetic markers and methodologies assessed, the best phylogenetic tree was obtained using the complete E2 phylogenetic marker using an ML approach (Fig. 2 and Supplementary Table S1). This tree was the best supported by the Shimodaira-Hasegawa’s test (Supplementary Table S1); as well, this topology yielded the best-supported bootstrap value for the internal node for the segregation between CSFV-genotype 1 (G1) and CSFV-genotype 2 (G2) (Fig. 2). The topologies solved from the smaller phylogenetic markers (5′-UTR (150 nt) and E2-partial (190 nt)) were significantly different from the topology obtained for the best phylogenetic tree selected (Fig. 2, Table S1). In addition, these two markers were unable to solve the monophyletic organization for the three main genotypes of CSFV. Low statistical support for internal nodes was also observed for these two markers (Fig. 2). The remaining phylogenetic markers (NS4B, complete E2 gene (1119 nt) and 5′-UTR-E2) yielded non-significant different topologies, excepting the phylogenetic marker 5′-UTR-E2 assessed under the ML methodology (see tree 14, Fig. 2). However, it is important to denote that only the topologies 7, 10, 11, 12, 13 and 15 could statistically support the divergence for the CSFV-G2 (Fig. 2). Based on all the results obtained from the phylogenetic markers evaluation, the marker E2 (1119 nt) was selected as the best phylogenetic marker for CSFV classification and evolutionary analysis.

### Phylogenetic analysis and classification cut-off

**Figure 3:**
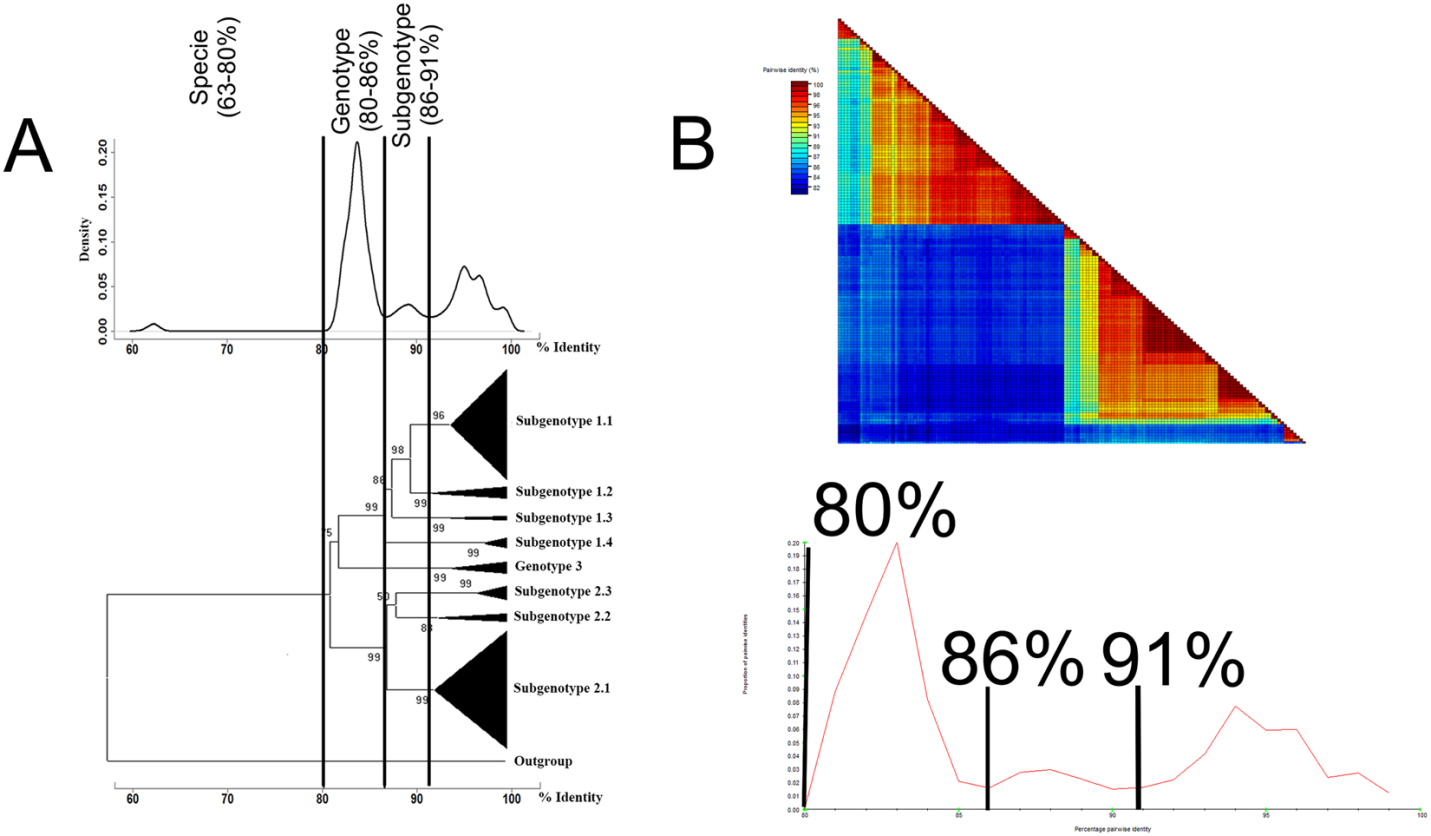
Frequency distribution of pairwise distance and clustering pattern for all lineages of CSFV using E2 gene sequences. (**A**) PASC results: the cut-off values for specie (63–80%), genotype (80–86%) and subgenotype (86–91%) differentiation were denoted, besides, a simplified tree deduced from the comparison of E2 gene sequences belonging to all lineages of CSFV and the Pestivirus Aydin/04-TR used as outgroup is shown. All the subgenotypes obtained were also denoted; (**B**) The SDT interface: a colour-coded pairwise identity matrix generated from all the 113 E2 gene sequences of CSFV included in the current study. Each coloured cell represents a percentage of identity score between two sequences (one indicated horizontally to the left and the other vertically at the bottom). A coloured key indicates the correspondence between pairwise identities and the colours displayed in the matrix. Pairwise identity frequency distribution plot is also shown. The horizontal axis indicates percentage pairwise. The cut-off values for genotype and subgenotype differentiation were also denoted.

The ML tree, based on the complete E2 gene from 115 CSFV sequences, identified the three main lineages (genotypes 1–3) and different sublineages (subgenotypes 1.1–1.4, 2.1–2.3) (Fig. 3A). The evolutionary divergence among the CSFV genotypes ranged between 0.153–0.175 (Supplementary Table S2), whereas the evolutionary divergence among the CSFV subgenotypes ranged between 0.08–0.115 and 0.101–0.122 for the genotypes 1 and 2, respectively (Supplementary Table S3).

Pairwise Sequence Comparison (PASC) analyses, based on the E2 gene from the 115 CSFV sequences, displayed a multimodal curve (Fig. 3A). Combining these results with a phylogenetic tree based on genetic distance approach, clear threshold values can be established. Thus, a threshold value of 91–86% of identity allows to separate all the subgenotypes of CSFV (Fig. 3A), whereas a threshold value of 80% of identity clearly shows segregation for all major CSFV genotypes, and the lowest value of 63% divides CSFV from the Pestivirus *Aydin/04-TR* (Fig. 3A).

Meanwhile the Sequence Demarcation Tool (SDT) analysis first yielded a color matrix in which three well defined groups of sequences could be easily identified (Fig. 3B). The pairwise plotting from SDT analysis showed the same cut-off values obtained using PASC analysis, therefore, 91–86% of identity to separate all the subgenotypes of CSFV and 80% of identity to segregate the three major CSFV genotypes was established (Fig. 3B).

### Evolutionary rates, tMRCA and phylodynamic analyzes

**Figure 4:**
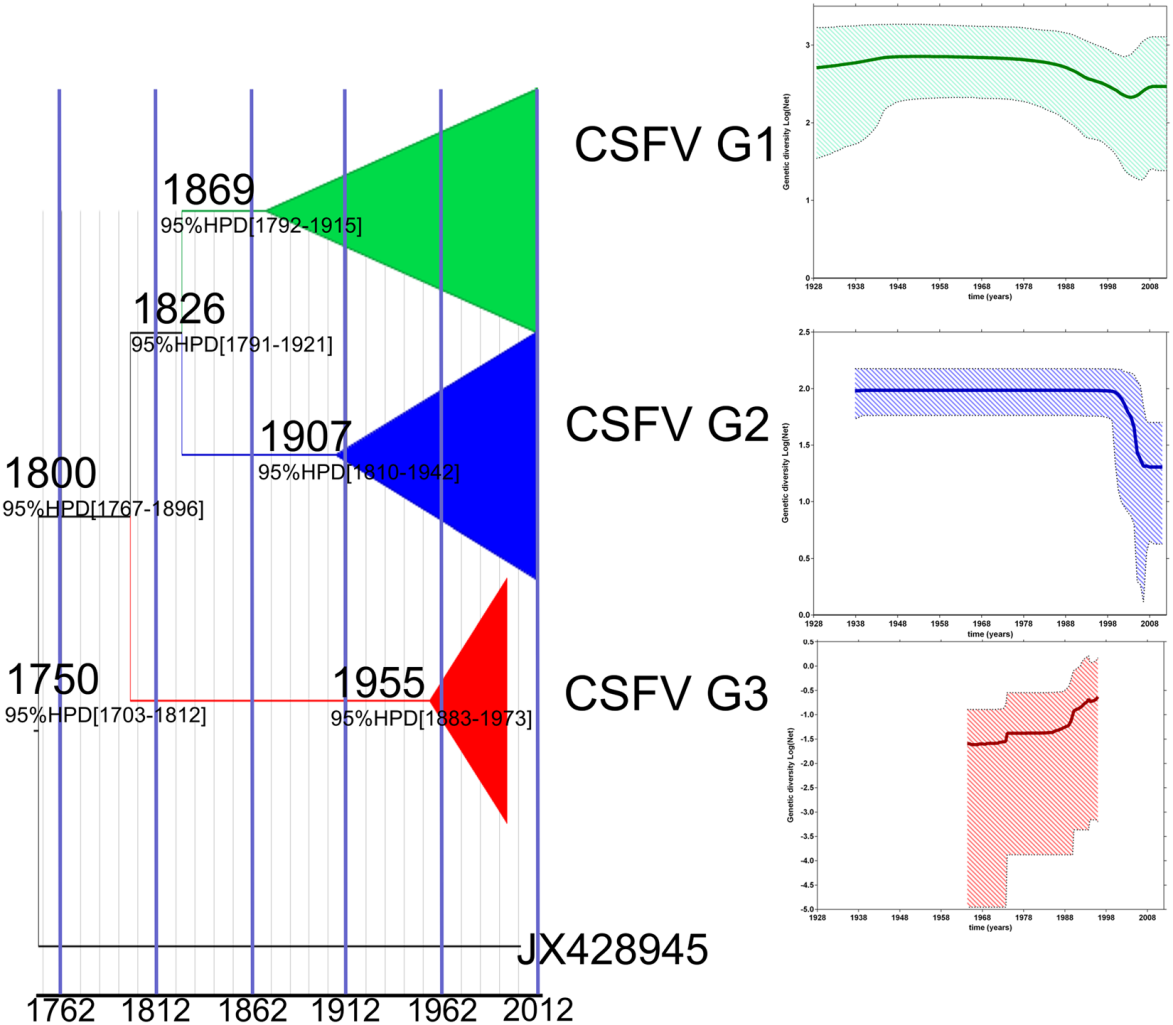
Comparative phylogenetic analyses and population dynamics of classical swine fever virus. Maximum clade credibility (MCC) tree constructed using BEAST program. For simplication, the main linages branches were collapsed. The branches belonging to the three main lineages of CSFV were highlighted in green for CSFV-genotype 1, in blue CSFV-genotype 2 and in red for CSFV-genotype 3. The most probable year for the MRCA within each lineage and the 95% highest probability density (HPD) were also denoted. The relative genetic diversity was estimated for each genotype (green: CSFV-genotype 1, blue: CSFV-genotype 2 and red: CSFV-genotype 3) by Bayesian skyline Plot using an exponential, uncorrelated clock model. The x-axis is in units of year, and the y-axis represents the logarithmic scale of Neτ (where Ne is the effective population size and τ is the generation time).

The path sampling (PS) and stepping-stone (SS) sampling methods analysis, based on the complete E2 gene phylogenetic marker, both showed an exponentially growing population size model with an uncorrelated exponential clock as the best-fitted model for our data (Supplementary Table S4). The estimated mean (95% highest probability density (HPD)) for the substitution rate of all populations of CSFV strains assessed was 7.09 × 10^−4^ (2.56 × 10^−4^–1.37 × 10^−3^) substitutions/site/year (Supplementary Table S5). However, at the genotype level, the three lineages showed different substitution rates. The estimated mean (95% HPD) of the substitution rate for genotype 1 (CSFV-G1) was 2.66 × 10^−4^ (7.98 × 10^−5^–5.02 × 10^−4^) substitutions/site/year; the estimated mean (95% HPD) of the substitution rate for genotype 2 (CSFV-G2) was 6.37 × 10^−4^ (4.53 × 10^−4^–8.45 × 10^−4^) substitutions/site/year; and the estimated mean (95% HPD) of the substitution rate for genotype 3 (CSFV-G3) was 1.31 × 10^−2^ (8.78 × 10^−3^–1.92 × 10^−2^) (Supplementary Table S5). The date Bayesian phylogenetic tree obtained for the global CSFV strains was characterised by a clear temporal structure; the oldest samples tended to fall closer to the root of the tree, while the most recent samples were located at the most distal tips. The mean tMRCA of the CSFV as a viral species was located at approximately year 1750 (95% HPD from 1703 to 1812) (Fig. 4). The diversification of the three main CSFV lineages was located at approximately 1800 (95% HPD from 1767 to 1896) (Fig. 4), whereas the mean tMRCAs of the three different CSFV genotypes (G1, G2 and G3) were framed in different dates, with the MRCA of G1 framed at approximately 1869 (95% HPD from 1792 to 1915), for G2 at around 1907 (95% HPD from 1810 to 1942) and for G3 at approximately 1955 (95% HPD from 1883 to 1973) (Fig. 4). Thus, the results obtained clearly suggest the emergence of CSFV-G1 first, followed by CSFV-G2 and finally CSFV-G3 (Fig. 4).

Demographic inference using the Bayesian skyline plot (BSP) model is summarised in Fig. 4. BSP essentially plots Neτ as a function of time. Neτ can be considered as a measure of relative genetic diversity that reflects the number of effective infections established by the virus. The BSP for the three main CSFV-lineages showed diverse patterns of Neτ, indicating different epidemiological behaviours among the viral populations (Fig. 4). For CSFV-G1, a maintenance in the Neτ from its emergence to the late ’80 s was observed, with a subsequent decrease from 1999 to 2003. From there it increased slightly followed by a constant Neτ value, suggesting stability in the diversity of this population for the last period (Fig. 4). Similarly, for CSFV-G2 Neτ was maintained from its emergence to the late ’90 s, with a sharp decline starting in 1998 followed by a constant value. This suggests that this lineage suffered a bottle-neck effect, affecting the stability in the diversity for this population (Fig. 4). Conversely, for CSFV-G3, an abrupt increase in Neτ from the emergence of this lineage (approximately in 1955) to 1998 was observed (Fig. 4), which proves an epidemic behaviour of this viral population during this period. However, no information on the following years could be obtained for this lineage.

### Structural model for E2 protein of CSFV

**Figure 5:**
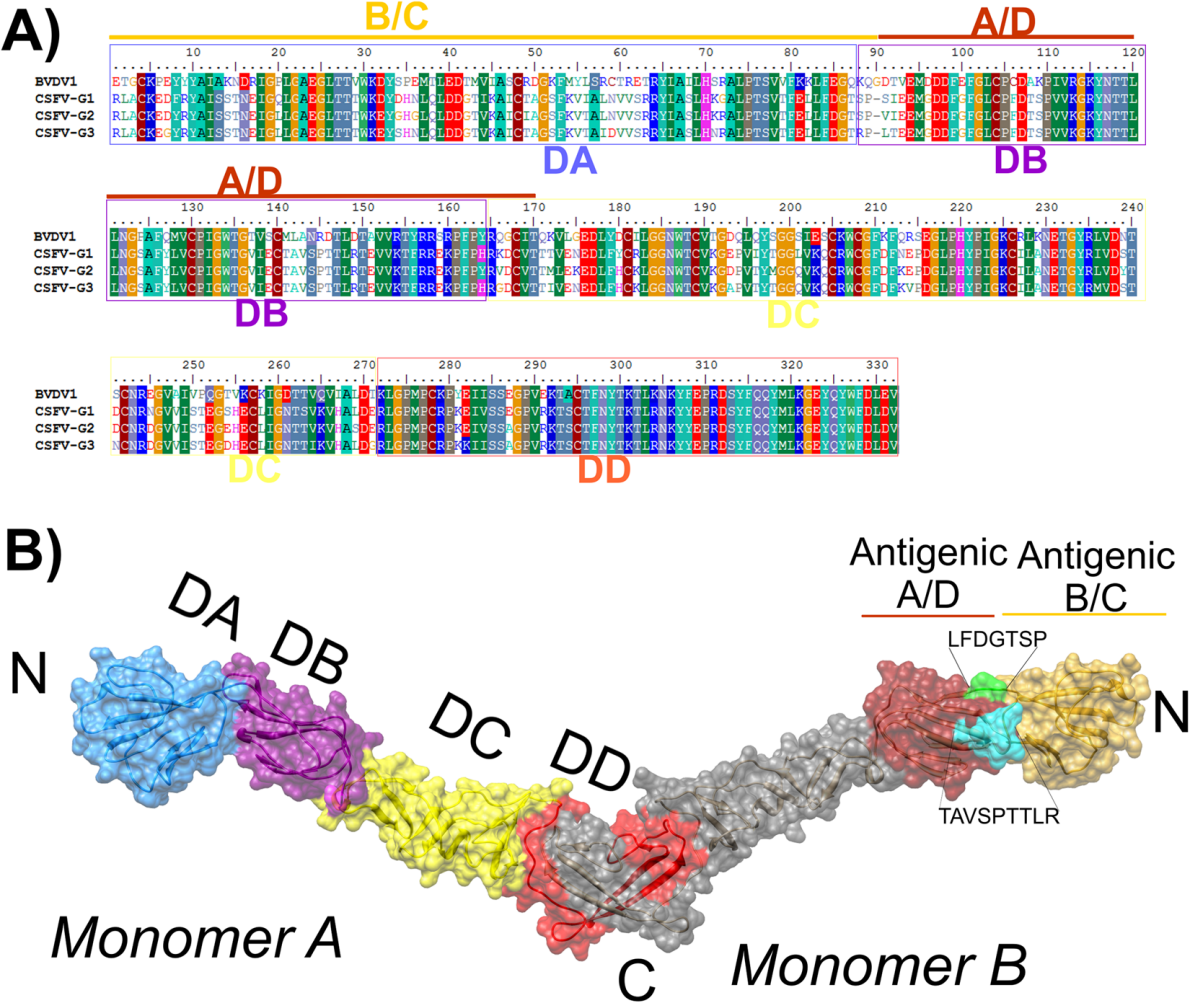
Cartoon and surface of the predicted model of E2 protein of CSFV. (**A**) Sequence alignment of the template (BVDV1 E2) with E2 proteins from three-representative sequences of the three genotypes of Classical Swine Fever virus (CSFV). The location of structural domains DA, DB, DC and DD on the amino acid sequence were colored in blue, green, yellow and red respectively, keeping the same pattern of the 3D representation. Likewise, the antigenic regions A/D and B/C were also denoted. (**B**) Folding of E2: structural domains were represented on monomer A starting from the N terminus colored in blue (DA), purple (DB), yellow (DC) and red (DD). Positions of the antigenic regions B/C (gold) and A/D (ruby red) were located on monomer B. The linear epitopes LFDGTNP (green) and TAVSPTTLR (cyan) were also represented.

At the time this study was undertaken, no crystal structure for the CSFV E2 protein was available. To better characterize this protein, a homology model was generated. The multiple sequence alignment obtained from three representative sequences of the three main CSFV-genotypes and the template sequence yielded 61% identity of sequence (IS) for the template/CSFV-G1 pair and 62% sequence identity for both the template/CSFV-G2 and the template/CSFV-G3 pairs (Fig. 5A). Since the IS for the three query sequences were higher than 50%, high quality structural models from homology method are expected^19^. Thus, a structural model for each CSFV genotype was obtained (Supplementary material, File S1). Six N-acetylglucosamine molecules were incorporated at positions N116, N185, N227, N448, N517 and N561 as post-translational modifications (Supplementary material, File S1). The Ramachandran plot (φ/ψ) distribution of the backbone conformation angles for each of the residues in the refined structure models revealed values in the favored region of approximately 98% (CSFV-G1 = 97.1%, CSFV-G2 = 96.8% and CSFV-G3 = 97.1%) and around 2% in the allowed region (CSFV-G1 = 2.3%, CSFV-G2 = 2.4% and CSFV-G3 = 2.4%). Outliers of 0.6%, 0.8% and 0.5%, for CSFV-G1, CSFV-G2 and CSFV-G3, respectively, were obtained.

The resulting model of each CSFV-genotype showed that the E2 monomer is an elongated molecule consisting of four domains: DA, DB, DC and DD, arranged linearly from N to C terminus (Fig. 5B). The DA domain (residues 1–87) contains a chain formed by six β-sheets with Ig-like folding (Fig. 5B). This domain contains a disulfide bridge at C4–C48. The DB-domain (residues 88–163) showed similar composition and folding to the DA-domain (Fig. 5A) with disulfide bridge at C103–C139. The antigenic domains B/C (residues 1–90) and A/D (residues 91–170) were located within these two structural regions (Fig. 5). The DC domain (residues 164–270) consists of a series of small β-sheet modules (Fig. 5). This domain has a disulfide bridge to the domain DB at C129–C167, and three additional intra-domain disulfide bridges at C180–C188, C204–C225 and C207–C241. The DC-folding and topology only share significant similarity with the template structure but not with other protein structures previously determined experimentally. Since domain DD is the most conserved domain among the Pestivirus genus, it showed identical shape with the template structure. The energy scores obtained from Discrete Optimized Protein Energy (DOPE) for the E2 structural model of CSFV-G1, CSFV-G2 and CSFV-G3 were −61334.27344, −60896.99609 and −60644.94922, respectively.

### Positive selection as an evolving force acting on CSFV-genotypes and subgenotypes

To test the hypothesis of positive selection on the complete E2 gene of CSFV, the site models and the branch site models implemented in the CODEML program of the PAML v4.7 software package were used^20^. The substitution rate ratios of non-synonymous (dN) versus synonymous (dS) mutations (ω) were calculated. The ω ratio should be 1 for genes subject to neutral selection, <1 for genes subject to negative selection, and >1 for genes subject to positive selection. In the site model, codon site models M1, M2, M7, and M8 were implemented using the LTR test to evaluate whether the site models assuming positive selection (M2a and M8) fit the data better than models without positive selection (M1 and M7)^20^. To test whether codon selection occurs among different CSFV-lineages, the branch site Model A, which allows for ω > 1 along foreground branches, was compared with the null Model A1, which only allows for ω ≤ 1 along foreground and background branches. This model comparison was made by labelling foreground branches of the three CSFV genotypes and all subgenotypes within every genotype.

**Figure 6:**
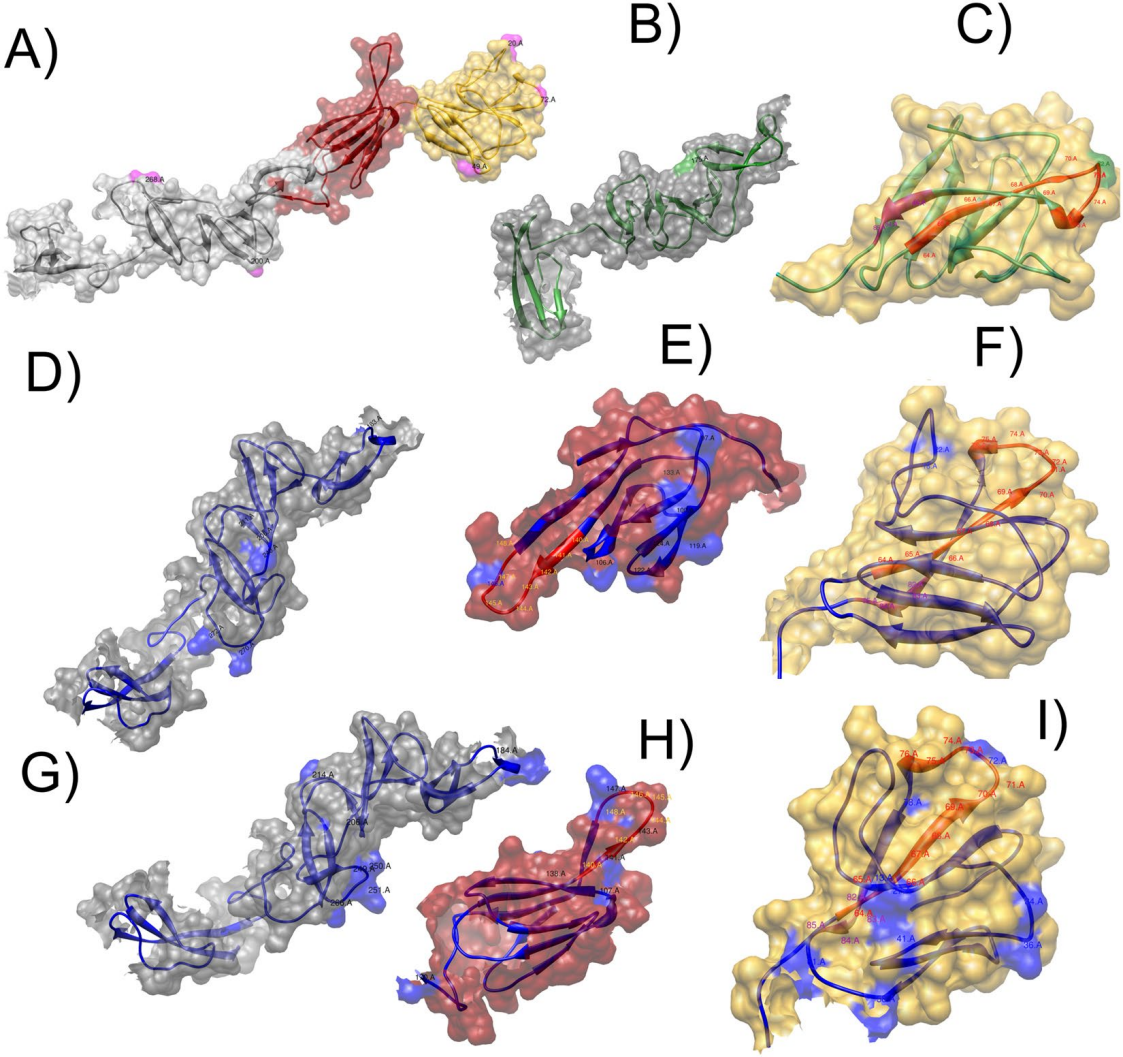
Mapping of positively selected sites on three dimensional structure of the E2 protein of CSFV. In all cases the surfaces for the antigenic domains B/C, A/D and the half C-terminal were represented in gold, ruby-red and gray respectively, the ribbon for the antigenic motives ^64^RYLASLHKKALPT^76^ and ^82^LLFD^85^ on the antigenic domain B/C were highlighted in red and pink respectively. (**A**) Positive selected sites identified by the site models M2a and M8 vs M1 and M7 respectively, for all 113 E2 gene sequences included in the study. All the sites were denoted in the ribbon structure and highlighted in pink on the protein surface. (**B**–**I**) Positive selected sites identified by the branches-site models (A1 vs A) for the three subgenotypes of CSFV under the influence of positive selection action. (**B**) and (**C**) CSFV-subgenotype 1.4, (**D**–**F**) CSFV-subgenotype 2.2 and (**G**–**I**) CSFV-subgenotype 2.3; the ribbon for the CSFV subgenotype 1.4 was represented in green and for the CSFV-subgenotype 1.2 was represented in blue. The sites detected under positive selection pressure by the branches-site model were denoted on the ribbon structure and colored on the surface (CSFV-subgenotype 1.4: green and CSFV-subgenotype 2.1 and 2.3: blue).

Based on the Bayesian posterior probabilities, five codon sites (positions: 20, 49, 72, 200 and 268) under positive selection pressure were identified from the M2 and M8 models (Supplementary Table S6). From these five codons, two were selected with a 0.01 significance level, whereas the remaining codons were detected at a 0.05 significance level (Supplementary Table S6). Three of the sites were situated in loops of the antigenic B/C domain and the remaining two were located in loops in the structural domain DB (Fig. 6A). In the branch site model, ω is allowed to vary both among sites in the protein as well as across branches on the tree, with the aim of detecting positive selection that only affects a few sites along particular lineages. The branches being tested for positive selection are referred to as the foreground branches, while the remaining branches on the tree are referred to as background branches. From the CSFV lineages tested, the results obtained showed that the action of positive selection caused the emergence only of the subgenotypes 1.4 with a value of ω2 = 7.471 (p < 0.01) (Supplementary Table S7). On this lineage, two sites were found: codons 72 and 175. The site 72 is located at the antigenic motif ^64^RYLASLHKKALPT^76^ in the B/C domain (Fig. 6C), whereas site 175 is in the loop of the structural domain DC (Fig. 6A). Even though the emergence of subgenotypes 2.2 and 2.3 was not caused by the action of positive selection (ω2 = 1, Supplementary Table S7), different codons were found under the action of the positive selection pressure in theses lineages that were statistically significant (Supplementary Table S7). In subgenotype 2.2 a total of 16 codons were found under positive selection (Supplementary Table S7): two of them in the B/C domain (Fig. 6F), eight in the antigenic domain A/D highlighting position 146 which was located at the linearized epitope ^140^TAVSPTTLR^148^ (Fig. 6E), and the remaining six in the structural domains DC and DD. These last sites (183, 206, 211, 240, 270 and 272 (Supplementary Table S7) are included in the mapped regions (Fig. 6D), which interact with cellular β-actin in the process of fusion and viral entry^21^. Meanwhile, in subgenotype 2.3, a total of 22 codons were found under positive selection (Supplementary Table S7): nine of them in the B/C domain (Fig. 6I) denoting position 72 at the antigenic motif ^64^RYLASLHKKALPT^76^, six in the antigenic domain A/D with position 147 located at the linearized epitope ^140^TAVSPTTLR^148^ (Fig. 6H), and the remaining seven in the structural domains DC and DD, involved in the process of fusion and viral entry (Fig. 6H). For the remaining CSFV-genotypes or subgenotypes, no additional codons under positive selection were obtained (Supplementary Table S7).

### Functional divergence analysis of the E2 protein of CSFV

**Figure 7:**
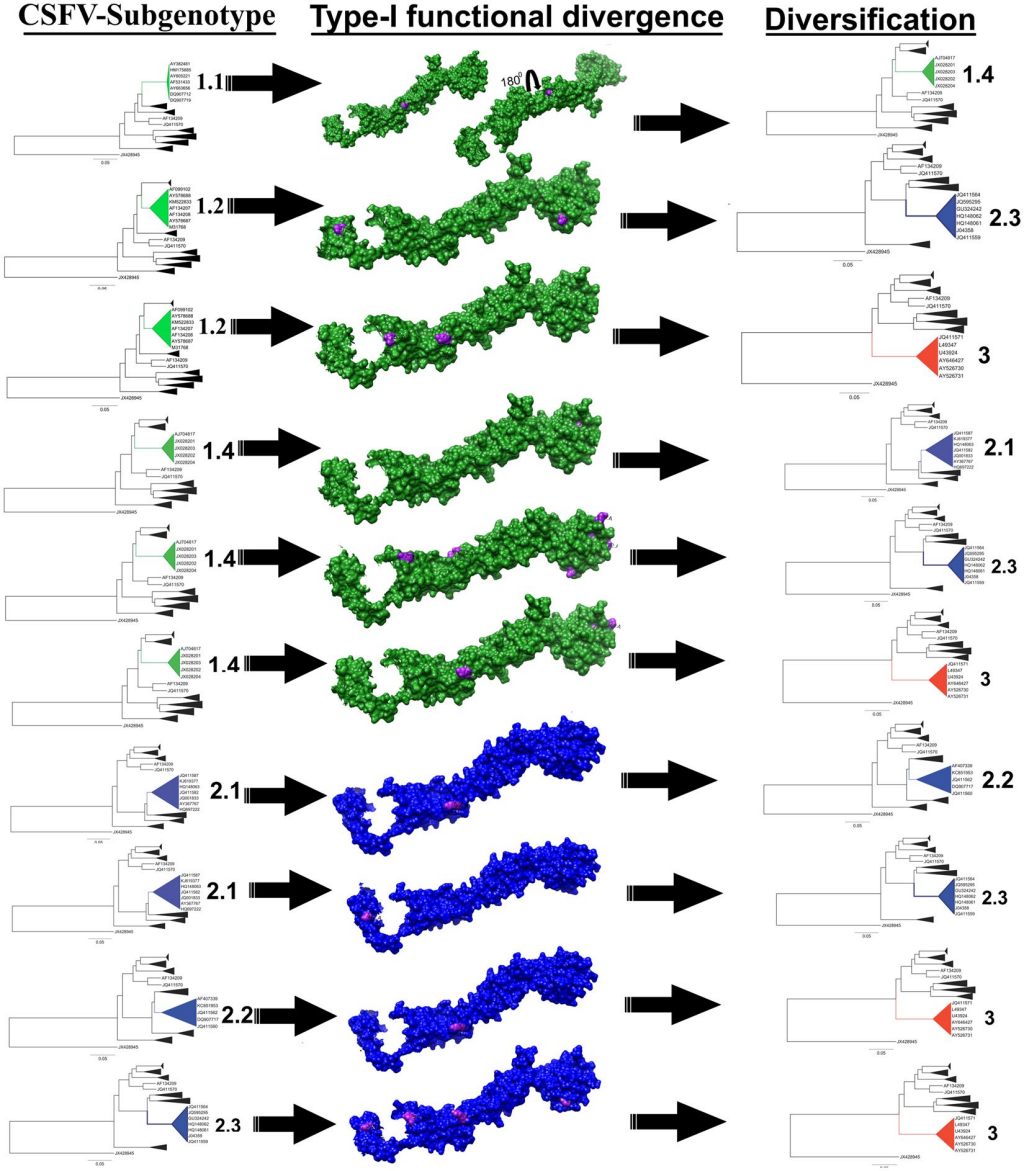
Mapping of functional divergence sites on the three dimensional structure of the E2 for the different subgenotypes of CSFV. Functional divergent selected sites were denoted in pink on the protein surface. The subgenotype involved in the funcional divergence type I were represented. The cluster was collapsed for simplification purposes. The sequence IDs belonging to each cluster involved in functional divergence episode were also denoted.

The results obtained indicate that the coefficients of Type-I functional divergence (θI) among some CSFV-subgenotypes and genotypes were statistically significant (p < 0.05) or strongly statistically significant (p < 0.01) (Supplementary Table S8). Hence, significant site-specific changes altered the selective constraints of CSFV subgenotypes, leading to subgroup-specific functional evolution after diversification of these subgenotypes. In addition, the critical amino acid residues responsible for functional divergence were identified by a combination of suitable cut-off values derived from the Qk and false discovery rate (FDR), which provides more statistical evaluations for predicted sites. A total of 13 sites were predicted through type-I functional divergence analysis (Supplementary Table S8). To analyse the possible role of these amino acid changes among the different clusters, the sites identified were mapped on the E2 protein structure together with the CSFV-tree representations (Fig. 7). Five sites were identified in the antigenic domain B/C with two sites (72 and 74) included in the antigenic motif ^64^RYLASLHKKALPT^76^. Addtionally, two sites in the antigenic domain A/D denoting the position 166 were included in the E2 host binding site ^141^AVSPTTLRTEVVKTFRRDKPFPHRMDCVTT^170^, six sites in the structural domain DC with the sites 192, 195, 253 and 268 involved in the process of fusion and viral entry, and two sites in the structural domain DD were identified (Supplementary Table S8 and Fig. 7).

### Cophylogenetic analysis

The procrustean superimposition plot of axes one and two, corresponding to patristic distances of species belonging to *Pestivirus* genus and their mammalian hosts, suggested four groups of host-parasite associations (Supplementary Fig. S1): One group consists of associations between BVDV1 and *Capreolus capreolus* and *Cervus elaphus* as well as an association between BDV3 and *Rangifer tarandus*; a second group concerns the association between BVDV1, bungowannah (PPB) and CSFV with *Sus scrofa*. This group is topologically similar to the third group, formed by species of BDV1, BDV2, BDV3, tunisian sheep virus (TSV), BVDV2, and Aydin associated with *Ovis aries*; the fourth group contained the species BVDV1, BVDV2 and pestivirus giraffe (PG) associated with *Bos Taurus*. The PACo analysis produced a residual sum of squares (m^2^_XY_) of 0.23925 with an associated permutational p < 0.05, clearly supporting the overall congruence.

**Figure 8:**
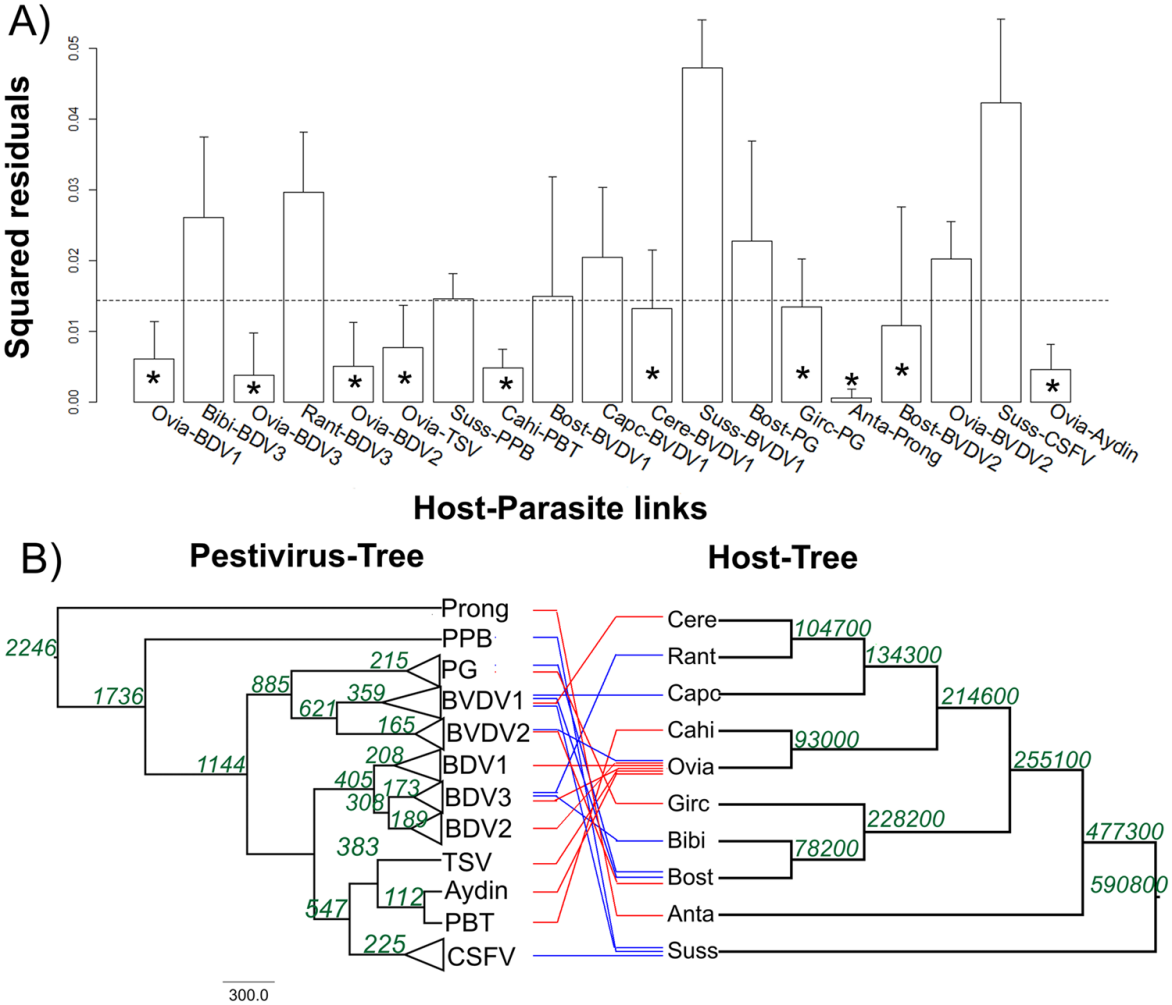
Virus-host evolutionary association in the Pestivirus genus. (**A**) Contributions of individual host-parasite links to the Procrustean fit: Jacknifed squared residuals (bars) and upper 95% confidence intervals (error bars) resulting from applying PACo to patristic. Asterisks identify links significantly supported. The median squared residual value is shown (dashed line). (**B**) Tanglegram indicating the associations between each pestivirus and its reservoir host. The numbers at the nodes indicate the divergence time for that node, as estimated using the BEAST softaware packge. Red lines represent host-parasite associations observed which were significantly supported by PACo and blue lines the unsupported links. Parasite (BDV1: border diseases virus 1, BDV2: border diseases virus 2, BDV3: border diseases virus 3, TSV: tunisian sheep virus, PPB: pestivirus bungowannah, PBT: pestivirus Burdur/05-TR, BVDV1: bovine viral diarrhea virus 1, BVDV2: bovine viral diarrhea virus 2, PG: pestivirus giraffe, Prong: pronghorn antelope pestivirus, CSFV: classical swine fever virus, Aydin: aydin pestivirus); host (Ovia: *Ovis aries*, Bibi: *Bison bison*, Ranta: *Rangifer tarandus*, Suss: *Sus scrofa*, Cahi: *Capra hircus*, Bost: *Bos Taurus*, Capc: *Capreolus capreolus*, Cere: *Cervus elaphus*, Girc: *Giraffa Camelopardalis*, Anta: *Antilocapra americana*).

The bar plots of squared residuals using patristic distances (Fig. 8A) indicated that the 10 links between *Pestivirus* genus and their related host contribute relatively little to m^2^_XY_ and thus likely represent coevolutionary links. Thus, the species BDV1-3, TSV and Aydin pestivirus, seem to have evolved with their host *Ovis aries* since their emergence. Similarly, the species Pestivirus Burdur/05-TR (PBT), BVDV1, PG, Pronghorn antelope pestivirus (Prong) and BVDV2 seem to have evolved with their hosts *Capra hircus*, *Cervus elaphus*, *Giraffa camelopardalis*, *Antilocapra Americana*, and *Bos Taurus*, respectively, since their emergence (Fig. 8A). The remaining relationships, including the one between CSFV and *Sus scrofa*, rejected the coevolutionary hypothesis. The reconciliation of the *Pestivirus* tree with the host tree revealed that a maximum of two cospeciation events might have occurred in their evolution (Supplementary Fig. S2). This reconciliation also contained six host switches and seven failures in diverge duplications (Supplementary Fig. S2). The switch of TSV from *Ovis aries* to the new host, *Sus scrofa*, is the most probable event causing the emergence of CSFV (Supplementary Fig. S2). The total cost for this reconciliation was 37. Based on both analyses (PACo and Jane analyses), the reconciliation among the different Pestivirus and their respective host was reconstructed. To test for consistency between the divergence times of each pestivirus species with those of their hosts, the tMRCA values for a range of ruminant Pestivirus and CSFV were estimated (Fig. 8B). We considered only the upper 95% HPD of these tMRCA estimates for a conservative comparison with the host divergence times. The tMRCA for CSFV was estimated around 225 years, whereas the divergence of *Sus scrofa* of the ancestral forms was estimated at approximately 590,000 years (Fig. 8B).

## Discussion

Despite recent progress in the understanding of CSF and its causal agent CSFV, this viral disease remains a major challenge for the scientific community^2^. In our opinion, it is imperative to gain deep insight into three relevant topics: (i) the mechanisms driving CSFV’s evolution and diversity, (ii) the role of *quasispecies* composition as viral determinants of virulence and (iii) the molecular mechanisms responsible for viral persistence in the host. Using *state-of-the-art* methodologies including phylogenetic inference, homology modelling, phylodynamic and host-virus reconciliation reconstructions, the current study was focused on deciphering the origin, genetic variability and evolutionary process of CSFV.

In a first approach, the reliability of the most commonly used phylogenetic markers to perform molecular epidemiology studies of CSFV^4^ was evaluated. Although complete genome sequencing for CSFV has been performed, which increased the total of complete genome sequences in public databases such as GenBank, this number remains limited since not all previously classified genotypes/subgenotypes are represented in those public domains. Hence, finding a highly reliable phylogenetic marker that is capable of reproducing the same phylogenetic information as the complete genome is an essential task. From the phylogenetic marker assessment, the complete E2 gene was found to be the best phylogenetic marker. This result was supported by different parameters previously described in Alfonso-Morales *et al*.^22^. Previous studies have observed that, with regards to the saturation of substitutions, if a dataset is saturated, phylogenetic reconstruction may be misleading due to the homoplasious signal^23^. Of all phylogenetic markers evaluated, only the region proposed by Paton *et al*.^24^ showed a saturation of substitutions phenomenon.

The power of a dataset is another critical factor to guarantee a reliable phylogenetic design. Phylogenetic noise from the fast-evolving sites can mislead phylogenetic inference. Identification of optimal levels of noise exclusion reduces the number of topologies that are not significantly worse than the optimal tree. This allows for a more robust inference of phylogeny and stronger conclusions about evolutionary character^25^. In this study, the phylogenetic noise associated to all larger phylogenetic markers (NS4B, E2-complete and 5′UTR-E2) showed lower values than the shorter markers (5′UTR and E2-partial). However, the lowest phylogenetic noise was shown by the E2-complete marker, with the same value as the complete CSFV genome. This strongly supports the use of the E2 complete region as phylogenetic marker. High consistency between specific regions and their whole genome in phylogenetic relationships can be considered as a good signature of a phylogenetic marker^26^. However, the best possible phylogenetic estimation can come from using robust inference methods allied with accurate evolutionary models and proper statistical support. A commonly used method for assessing the robustness of a tree is the non-parametric bootstrapping. Nevertheless, bootstrap values are more suitable for examining small parts of the tree (one or two key branches) rather than the whole tree^27^. Meanwhile, non-parametric bootstrap tests based on likelihood ratio tests (LRTs), such as the Shimodaira and Hasegawa test, provide a straightforward means to decide which topologies best fit the data under analysis. These type of tests consider all possible topologies and make the proper allowances for their comparison with the ML topology derived from the same data^27^. In this study, all parameters assessed and both statistical support analyses (parametric and non-parametric) strongly supported the E2-complete marker as the best phylogenetic marker for CSFV, capable of reproducing the same phylogenetic information as the complete viral genome.

Given the reliability of the E2-complete marker, this marker was used to establish a cut-off for classification purposes in CSFV using PASC and SDT. Both PASC^28^ and SDT^29^ are powerful methods used for viral classification that use pairwise genetic identity calculations. In addition, PASC and STD methods have also been widely employed to determine the criteria for sublineage classification that is accepted by the International Committee on Taxonomy of Viruses (ICTV). The cut-off value obtained for both methods to classify a new subgenotype of CSFV was 91-86% of sequence identity. A recent report has proposed the circulation of ten new subgenotypes of CSFV in China^8^ using the E2-complete marker for phylogeny inferences; however, the percentage of maximum inter-lineage nucleotide variations among several of these subgenotypes was lower than 9% (eg: 2.1h-2.1b 93.6-96.1% of identity of sequences (see Table 2 in Gong *et al*.^8^)). In addition, the node of the phylogenetic tree where the subgenotypes 2.1a,b,h,i,j diverge from the 2.1g was only supported by a 46% bootstrap value, which indicates that this segregation was just a random topology and not statistically supported (see Fig. 2 Gong *et al*.^8^).

This analysis clearly demonstrated that subgenotypes 2.1 a,b,g,h,i and j are not distinct enough to be regarded as new subgenotypes. As proposed in the current study, we established a reliable cut-off for classification of CSFV, based on two methods widely accepted by ICTV and the scientific community with the aim to avoid further misclassifications of this viral agent. It also intends to standardize nomenclature for this viral specie and encourage other authors to follow the statement of Strengthening the Reporting of Molecular Epidemiology for Infectious Diseases (STROME-ID)^30^.

The emergence of CSFV as viral specie and the expansion of the CSFV-lineages were also estimated. The MRCA of CSFV emerged approximately between 1703–1812 with a consequent diversification approximately between 1767–1896. The first evidence of a swine illness resembling CSF was described in U.S during the 1830s. However, at that time, the illness was not considered a significant problem. Losses were generally confined to a single animal at a time, with a first outbreak of the condition attributed to CSF in Ohio in 1833. Only 10 outbreaks in 10 different states were reported through correspondents from 1833 to 1845^31^. However, in the U.S, CSF rapidly changed with 173 CSF-outbreaks recorded in 22 states between 1856 to 1860. By 1887, the disease had been reported in 35 states, from Maine to Texas to California^31^. In Europe, the disease was first described in 1862 as “swine fever” in England, “Swinpest” in Sweden and “pneumo-entérite infectieuse” in France. It later spread to Denmark and Germany in 1887^32^. The results obtained in the current study coincide with the historical reports of CSF outbreaks. The previous estimation of CSFV emergence framed it approximately 2770.2 years ago^13^ without any historical or epidemiological evidence. Several factors could bias this result, including the genomic region used to estimate the tMRCA, the number of sequences and the reliability of the phylogenetic methods accomplished^13^. A critical analysis can show that Kwon *et al*.^13^ used the Bayesian factor to select the evolutionary model even though PS or SS were described as the best estimators^33^. Additionally, these authors only established a confidence value of ESS >100 instead of an ESS >250 which has been recommended to avoid false estimations^34^. The sum of all these changes may have affected the tMRCA’s reliance on the estimation for CSFV emergence and disconnected the origin of the virus from the historical context.

CSFV appears to exhibit significant heterogeneity in the rates of evolutionary changes among genotypes. All of the evolutionary changes were within the evolutionary rate of RNA viruses^35,36^. The differences found in the evolution of the three CSFV-genotypes could be a consequence of the diverse times of emergence. Thus, because CSFV-G1 showed the lowest evolutionary rates, it could possess better adaptation to the host, being CSFV-G3 the least adapted to the host. Regarding the phylodynamic results obtained for the population of CSFV, different structures for each CSFV-genotype were obtained. The stability in the effective population size (Nτe) for CSFV-G1 during its first period of circulation clearly reflects an endemic situation for this genotype in the regions where it was present. CSFV-G1 has remained endemic in the American continent for several decades^4^. In the U.S, CSFV-G1 circulated until 1976^3^, in Chile and Argentina until 1996^3^ and 1999^37^ respectively, and in Mexico it was present until 2009. However, in countries such as Brazil, Colombia, Honduras, Nicaragua, Guatemala, Dominican Republic, Haiti and Cuba, epidemic or endemic CSF-outbreaks are still reported^14,38^. Moreover, CSFV-G1 has been recently reported causing outbreaks in China, Russia and North-Eastern India^4^. Thus, the slight decline in the genetic diversity of CSFV-G1 from the late 90′s, with its lowest point in diversity in 2003, seems to be related to the control measures used against CSFV in America, especially after the continental plan from FAO in 2000^39^ for the eradication of CSFV. However, the fact that the disease remains endemic in some countries leads to the preservation of its genetic diversity. CSFV-G2 has been considered the most prevalent CSFV-genotype over the past two decades^4^. This genotype has been found mainly in Europe, Asia and Africa^4^. Therefore, the high genetic diversity found in the BSP analysis could be the consequence of the endemic situation related to this genotype. The sudden loss of diversity, a population bottleneck that occurred between 1998–1999, seems to be related to the stamping-out policy applied in Europe during the devastating CSF epidemic of 1997–1998 that affected Germany, the Netherlands, Italy, Spain and Belgium, caused by a virus strain of CSFV-G2^40^. On the other hand, the endemic circulation of CSFV-G2 in pigs in areas of China^4^ and India^4^, as well as in wild boar in Europe, can contribute to the maintenance of genetic diversity in this genotype. Meanwhile, the CSFV-G3 lineage had an abrupt increase in Neτ from its emergence (approximately in 1955) to 1998 (Fig. 4), which corresponds to an epidemic behaviour during this period. However, no further information about the following years is available for this lineage.

Another aspect addressed in the current study was the action of the evolutionary forces that lead to the genetic variability and the evolution of CSFV. Our results strongly evidenced that the E2 protein of CSFV is under positive selection pressure. Previous studies have shown this selective force acting on different sites of the E2-glycoprotein of CSFV^14,17,18,41^. In the B/C domain, several sites under positive selection have been described: Tang *et al*.^41^ reported the sites 72 and 75; Perez *et al*.^14^ found the positions 34, 36, 49, 72 and 49; Ji *et al*.^17^ detected the sites 34 and 49 whereas Hu *et al*.^18^ identified the sites 17, 34 and 72. In the current study, using site detection models and branches-site models, a total of 13 sites in B/C domain were found to evolve under positive selection pressure. Four of them (34, 36, 49, 72) have been previously linked to a decrease in the virulence of the field strains and promotion of viral escape from the host immune response^14,17,18^. The remaining nine sites (13, 18, 20, 22, 27, 41, 58, 61, 78) are reported for the first time, and were located in the loops that connect the antiparallel β-strand structures in the most exposed region of the viral surface (Fig. 6C,F and H). Hence, these sites could also play a role in the antigenic specificity of CSFV or in leading the evasion of the host immune response against CSFV. Nonetheless, the biological significance of these sites needs to be further characterized. Employing branches-site models, several sites under positive selection pressure were detected in the A/D antigenic domain. In this antigenic domain, no previous reports have described the action of this evolutionary force. However, Leifer *et al*.^42^ reported the presence of CSFV-escape variants generated under selective antibody pressure with monoclonal antibodies, showing amino acid changes in the immunodominant and highly conserved linear TAV-epitope (^139^C^**140**^ **TAVSPTTLR** ^**148**^TEVVK^153^) located in the A/D domain. This change demonstrated by the parental strains, which evolved after subsequent passages under selective antibody pressure, could result in a small fitness advantage^42^. The biological evidence showed by Leifer *et al*.^42^ could support the outcome of the current study detecting the action of the positive selection pressure on the sites found on the linear TAV-epitope 141, 143, 147 and for the spatially near residues 107 and 138 (Fig. 6H). However, to define the functional importance of the remaining sites, additional studies will be required. Studies focused on sites 100, 119, 122, 124, and 133 which were found together on the same protein surface, will be particularly important since these sites may potentially create an undescribed epitope (Fig. 6H). On the carboxyl-terminal half of the E2 protein, 13 sites influenced by the positive selection pressure were determined. From these sites, the position 200 has been previously described under positive selection pressure^18,41^ and has been linked to the attenuation of highly virulent CSFV-strains^43^. The residue 240 was determined by Hu *et al*.^18^ as a positively selected site but no biological characterization has been conducted yet. In our study, in addition to these two previously described sites, the positions 183, 184, 206, 211, 214, 249, 250, 251, 266, 270, and 272 were determined as positively selected. A recent study has located two functional domains of E2 on the regions 182–261 and 262–341 that interact with β-actin, a major cytoskeletal protein involved in many cellular processes including cell adhesion, cell migration/movement, cytokinesis and endocytosis/exocytosis^44^. Thus, the novel residues detected under positive selection pressure on the carboxyl terminal domain could play a role in the early replication stages or in tropism of CSFV. Nevertheless, further studies to verify their potential biological functions will need to be accomplished.

From the branches-site model, only the CSFV-subgenotype 1.4 was found under episodic positive selection. Interestingly, this CSFV-subgenotype has been restricted to Cuba^7^. Several factors could have conditioned this event in the CSFV evolutionary pattern. First, it is important to consider that CSFV has circulated in Cuba since 1930^3^ (probably coming from the USA with the introduction of the *Yorkshire* breed (http://repositorio.utc.edu.ec/bitstream/27000/683/1/T-UTC-0544.pdf)). Since then, controls policies have been put in place that include a vaccination campaigns using first a crystal violet vaccine and later a locally produced modified live vaccine containing a lapinized CSFV Chinese strain^3^. During the establishment and improvement of the Cuban pig industry, three relevant swine importations took place: in 1968 from Canada, the introduction of the *Duroc Jersey* breed; in 1989 from United Kingdom to introduce the *Landrace* breed (http://repositorio.utc.edu.ec/bitstream/27000/683/1/T-UTC-0544.pdf); and in 2005 another importation from Canada^45^. The fact that these movements of pigs to Cuba were from countries with a CSF-*free status* combined with the fact that Cuba is an island suggests that spatial segregation is responsible for the viral population in this country. Previous molecular epidemiology studies have shown that all Cuban CSFV isolates share a common origin with the CSFV “Margarita” strain (Cuban viral strain isolated in 1958) as ancestor^14,46,47^. Thus, these studies also support the premise of segregation for the Cuban CSF-viral population. Another aspect to consider is that the Cuban viral population has been under continuous pressure exerted by the vaccine. Pérez *et al*.^14^ showed that the inability of the Cuban vaccine used since 1962 to induce sterile immunity has lead to a bottleneck effect on the viral population. This event has also been linked to a potential adaptive advantage for viral field strains. The resulting viral progeny has shown to cause less severe clinical signs in the animals affected^14,48^, which has been associated with persistent infection^49^ and a decrease in the virulence of the circulating strains^15,16,48^. Thus, both spatial segregation and the selection pressure exerted by the vaccine seem to drive the evolutionary pattern of CSFV-subgenotype 1.4 as lineage positively selected.

Our results also showed that the protein E2 suffered functional divergence during the evolutionary process and diversification of CSFV. The residues found under Type-I of functional divergence play different roles in the viral pathogenesis such as antigenicity and viral entry and attachment^21^. Therefore, changes in these positions might be associated with functional adaptiveness of E2, promoting a change in the viral tropism, rate of infectivity, or escape from the host immune system response. Li *et al*.^49^ recently described the action of functional divergence on different residues of the E1 and E^rns^ proteins of CSFV. The positions found were linked to functional domains playing a role blocking the activation of the innate immune system, as well as in the viral attachment and entry into host cells^49^. Surprisingly, these authors didn’t find any E2 protein residues under the influence of functional divergence. However, taking into account that the action of positive selection pressure on the E2 protein has been previously described^14,17,18,40^, it is highly probable that those mutations fixed by positive selection can promote new functions for this viral protein. The differences in results obtained in the current study and the results described by Li *et al*.^49^ could be due to the sequence datasets used, cut-off values established, implementation of the false discovery rate (FDR) analysis, among others.

The evidence of recent positive selection on CSFV alternatively suggests that this viral agent has recently emerged in pigs. To our knowledge this is the first report approaching a cophylogenetic analysis for CSFV. Based on both analyses (PACo and Jane analyzes), several host switches for different *Pestivirus* members, including CSFV, were revealed. In fact, Geoghegan *et al*.^50^ have recently described in a study including a total of 19 viral families, that RNA viruses show a high frequency for host switching events. Considering that CSFV is an RNA virus with high rates of evolutionary change, this characteristic could confer more rapid adaptation to new environments, which, coupled with the frequency of exposure to new hosts, could facilitate host-switching. Our results indicated that CSFV emerged due to a switch of Tunisian sheep virus (TSV) from the host *Ovis aries* to the new host, *Sus scrofa*, in an event that occurred around 225 years ago. From the analysis performed, this event could have taken place around 1790. A historical review of the origin and development of the Animal Industry of the United States described the importation of the first Tunis sheep as a gift from the Bey of Tunis to Judge Richard Peters of Pennsylvania around 1799. Judge Peters, who was a practical farmer and the founder and first president of the Philadelphia Agricultural Society^51^, spread the Tunis sheep breed using native ewe, and their popularity continued to grow until approximately 1829 when they were one of the predominant breeds in this country^51^. As a characteristic of this period, mixed-farms allowed this new introduced specie to interact with the native pigs. Hence, these two different hosts, habiting the same geographic region, increased their interactions, facilitating the cross-species transmission of TSV, which could have resulted in the emergence of CSFV. The results obtained here using cophylogenetic analysis and a time-calibrated phylogenomic approach are in concordance with the historical, geographical and epidemiolocal evidence regarding the emergence of CSFV.

## Conclusions

Our study provides novel and significant insights into the origin, diversification and evolutionary process of CSFV. The E2-complete marker was selected as the best phylogenetic marker for CSFV capable of reproducing the same phylogenetic and evolutionary information as the complete viral genome. By using this molecular marker, a reliable cut-off to establish an accurate classification of CSFV at genotype and subgenotypes levels was determined. Based on tMRCA reconstruction and cophylogenetic analysis it was proposed that CSFV emerged approximately 225 years ago as result of the Tunisian Sheep Virus host-jumping from its natural host to swine. CSFV emergence was followed by a genetic expansion in three main lineages due to the action of the positive selection pressure and the functional divergence as the main natural forces driving the genetic diversity and the evolution of the virus. In addition, in our study, a structural 3D model for the main viral immunogenic protein (E2) was obtained, which allowed us to map those residues involved in different process of the viral infection, antigenicity, pathogenesis and other unidentified functions. This model could also be useful to identify novel epitope targets for new vaccine candidates or new diagnostic assays in the future.

## Materials and Methods

### Sequences dataset and multiple alignment

Different sequence datasets were organized: (i) to assess the reliability of the different phylogenetic markers^13,24,52,53^, which have been commonly used to study the diversity and the evolutionary process, 65 non-redundant sequences of the complete genome of CSFV available at GenBank Database (http://www.ncbi.nlm.nih.gov/) were used (Supplementary Table S9). From these sequence datasets, different subsets were prepared: one containing the alignment of the entire genome of CSFV and the others containing only the phylogenetic marker regions to be assessed; (ii) to determine which evolutionary forces drive the CSFV-lineages evolution, a dataset that included all the sequences available at GenBank for the best phylogenetic marker selected was used (Supplementary Table S10). The evolutionary history of CSFV lineages was estimated by a time-calibrated phylogenomic approach, which estimates rates of nucleotide substitution per site, per year and the time to the Most Recent Common Ancestors (tMRCA) of specific genotype. Only sequences with a known year of collection were included (Supplementary Table S10); (iii) to infer the divergence times and probable origin of CSFV in relation to other *Pestivirus*, all sequences of the E2 gene were collected from GenBank and aligned with the sequence of CSFV (Supplementary Table S11). It has been suggested that CSFV have strictly coevolved with their swine host species about 8000 years ago^13^. To test this hypothesis, complete mitochondrial cytochrome b gene sequences were collected from GenBank for those vertebrate species considered to be reservoir hosts of distinct *Pestivirus* (Supplementary Table S12). In all cases, the alignments of the sequences were performed using the algorithm Clustal W included in the program BioEdit Sequence Alignment Editor^54^.

### Model selection

The software jModelTest 2.0 was used to estimate the best-fit model using the Akaike and Bayesian information criteria (AIC and BIC)^55^. The best-fit models for the complete genome and all phylogenetic markers were selected and used for phylogenetic analysis.

### Phylogenetic analysis

To remove sequences with a possible recombinant event from the alignment of all sequence datasets, searches for recombinant sequences and crossover regions were performed using Geneconv, RDP, MaxChi, Chimera, BootScan, SiScan, 3Seq and LARD, all implemented in RDP3 Beta 4.1^56^. Programs were executed with modified parameter settings determined according to the guidelines in the RDP3 manual for the analysis of divergent sequences (available upon request). Recombinant sequences were tested with a highest acceptable p value of 0.05, and Bonferroni’s multiple comparison correction was used. Analyses were conducted twice to ensure the repeatability of results.

Phylogenetic relationships of the CSFV strains using the complete genome and all the selected phylogenetic markers were analyzed using the Neighbour Joining (NJ), Bayesian Inference (BI) and Maximum Likelihood (ML) methodologies as previously described in Pérez *et al*.^44^. The sequence JX428945 belonging to the *Pestivirus* strain *Aydin/04-TR*, which has been recently reported as the closest known *Pestivirus* relative of CSFV^57^, was used as outgroup.

### Comparison of topologies

All topologies were tested by the Kishino and Hasegawa test (K–H)^58^ and the Shimodaira–Hasegawa test (S–H)^59^, which computed the log-likelihoods per site for each tree and compared the total log-likelihoods for each proposed topology, using the PAMLv4.7 program^20^. Ten thousand replicates were performed using the K–H and S–H topologies tests by re-sampling the estimated log-likelihoods for each site (RELL model). Finally, the trees selected were visualized by FigTree v1.1.2^60^.

### Evaluation of the phylogenetic marker selected

The evaluation of the quality and reliability of every phylogenetic marker was performed following the methodology developed by our research group and recently described in Alfonso-Morales *et al*.^22^, briefly.

#### Evaluation of the substitution saturation

The loss of phylogenetic information due to substitution saturation was evaluated comparing the complete genome of CSFV sequences and the phylogenetic markers. The level of saturation was studied by plotting the pairwise number of observed transitions and transversions versus genetic distance. In addition, substitution saturation was evaluated with Xia’s test. All these studies were performed using the DAMBE program^23,61^.

#### Likelihood mapping

The phylogenetic signal of each sequence dataset was investigated by the likelihood mapping analysis of 100,000 random quartets generated using TreePuzzle. In this strategy, if more than 30% of the dots fall in the center of the triangle, the data is considered unreliable for phylogenetic inference purposes^62^.

### Evaluation of the phylogenetic-relationship reconstruction

To assess which phylogenetic marker contains the adequate phylogenetic signal to reduce cost without losing valuable information, topologies using complete genome of CSFV (*gold standard*) and the phylogenetic markers were constructed following the methodology described above. All topologies obtained using all datasets were compared as described above.

### Substitution rates, time-scale of evolutionary history and phylodynamic analyzes

The datasets including *Pestivirus* genus members, vertebrate hosts and CSFV sequences were used to generate the BEAST input file by BEAUti within the BEAST package v1.8.1^34^ (freely available at http://beast.bio.ed.ac.uk). Rates of nucleotide substitution per site, per year and the tMRCA were estimated employing a Bayesian MCMC approach. The model selection was performed by estimating model marginal log-likelihood through the path sampling (PS) and stepping-stone (SS) sampling methods described by Baele *et al*.^33^. The estimation of model marginal log-likelihood through the PS and SS for the twelve coalescent demographic models included parametric models (constant population size, exponential growth and logistic growth) and nonparametric models (Bayesian skyline plot, BSP) with strict, uncorrelated lognormal distribution (UCLD) and uncorrelated exponential distribution (UCED) relaxed molecular clocks were calculated (Supplementary Table S4). Rates of nucleotide substitution per site, per year and the tMRCA were also estimated. In addition, Bayesian skyline plot for CSFV genotypes to infer the population dynamics in terms of changing levels of relative genetic diversity (Neτ) through time was performed. For BSP analysis data was collected and plotted using *Graphpad Prism* software 6.0 (1992–2007, Graphpad Prism software Inc.).

In all cases the MCMC chains were run for 100 million generations, in order to obtain an ESS >250, and the first 10% trees were discarded as “burn-in”, as recommended by the BEAST package Manual^34^ (freely available at http://beast.bio.ed.ac.uk). Convergence was assessed by estimating the effective sampling size (ESS) after a 10% burn-in, using Tracer software version 1.5 (http://tree.bio.ed.ac.uk/software/tracer/). The trees with maximum log clade credibility were selected and visualized by FigTree v1.1.2^60^.

### Classification analysis using PAirwise Sequence Comparison and Sequence Demarcation Tool

International Committee on Taxonomy of Viruses (ICTV) has endorsed, amongst other phylogenetic and biological criteria, the use of genome-wide nucleotide or amino acid sequence identity thresholds for the classification of novel virus isolates (according to ICTV proposals published since the 8th ICTV Report; http://ictvonline.org/). To test the reliability of the lineages defined for CSFV and establish a taxonomic cut-off, PAirwise Sequence Comparison (PASC)^28^ and the recently developed Sequence Demarcation Tool (SDT)^29^ were used. On the one hand, PASC is a widely-accepted method in virology, which is based on the histogram of pairwise differences among sequences, also known as mismatch distribution. Thus, PASC was performed using mean distances among and within lineages calculated by means of the Mismatch Distribution function included into PEGAS software available in R Package^63^. On the other hand, SDT is functionally similar to PASC in that it objectively applies a robust Needleman-Wunsch (NW)-based pairwise alignment approach with a pairwise identity calculation that ignores alignment positions containing indel characters, but this new algorithms is not restricted to using predefined sets of sequences and it has been geared specifically to the objective taxonomic classification within the context of ICTV endorsed pairwise identity based strain, species and genus demarcation thresholds. Thus, SDT analysis was performed for the dataset of the CSFV sequences following the SDT Manual^29^.

### Positive selection pressure for specific site and branches-site

Positive selection analysis from the complete E2 region was conducted using several models available in the CODEML module of PAML 4.7 software package^20^. Different values of non-synonymous/synonymous dN/dS rate ratio (ω parameter), were considered, according to the user guide manual. To avoid false positives, the models used to detect sites under positive pressure were contrasted with models used to detect neutral selection^64,65^, only cases in which the likelihood ratio test (LRT) result was significant were considered. The Bayes Empirical Bayes (BEB) calculation of posterior probabilities for site classes was used to calculate the probabilities of sites under positive selection^38^. Details of each model are as follows. Model M0 allows for a single ω value across the whole phylogenetic tree at all sites. Subsequent models allow ω to vary at different sites. Model M1a (nearly neutral) allows for two rates of ω to vary between 0 and 1, while Model M2a (positive selection) is the same as Model M1a but allows for an additional rate of ω to be >1. Model M8a assumes a discrete beta distribution for ω, which is constrained between 0 and 1 including a class with ω = 1, similar to Model M8 which allow the same distribution as M8a but has an extra class under positive selection with ω > 1. Branch-site tests, using pre-specified branches are hypothesized to have occurred (foreground branches), were made with the null Model A1. This allows ω ratios to vary among sites and among lineages, and it also provides two additional classes of codons with ω = 1 along pre-specified foreground branches, while restricting ω to be ≤1, on background branches. The alternative Model A allows ω to vary between 0 and 1, be equal to 1 for all branches, and has two additional classes of codons under positive selection with ω > 1 along pre-specified foreground branches while restricting ω to either 0–1 or ω = 1 on background branches.

For all LRTs, the null model is a simplified version of the selection model, with fewer parameters, and is thus expected to provide a poorer fit to the data (lower maximum likelihood). The null models (M1a, M8a, and A1) do not allow codons with ω > 1, whereas the selection models (M2a, M8, and A) are alternative models that allow for codons with ω > 1. The significance of the LRTs was calculated assuming that twice the difference in the log of maximum likelihood between the two models was distributed as a χ2 distribution with the degrees of freedom (df) given by the difference in the number of parameters in the two types of models^66^.

### Comparative modeling

The primary sequences (accession codes: Q5ZN69, Q06DW2 and I3QFN7) of CSFV E2 genotypes 1, 2 and 3 were retrieved from the UniProt KB database (http://www.uniprot.org). The structure of BVDV1 glycoprotein E2, (PDB: 2YQ2, PH = 8)^67^ was used as the template. All the sequences were imported into the ClustalX program^68^ for multiple sequence alignments (MSA). Sequences alignments were visually examined and manually adjusted by using Seaview 4.6.1^68^ in order to find the conserved patterns of cysteines involved in disulfides bridges, N-glycosylation sites, hydrophobic patches (C-terminal rich in phenylalanine and tyrosine residues) and linear epitopes. The resulting alignments (considering as a pair E2 sequence for each genotype and the template) were used to generate homology models by a Modeller 9.17 (http://salilab.org/modeller/) python script and graphically inspected with the user interface program UCSF Chimera^69^ (http://www.cgl.ucsf.edu/chimera/). Thus, for each CSFV genotype, the 3D-structure for the E2 protein was built as glycosylated homodimer following the instructions of the Modeller manual^70^. Briefly: the structures were determined using the BLK (‘.’) residue type in the alignment for both template and model sequences in order to copy the N-acetyl-D-glucosamine residues as a rigid body into the model and adding one chain break (‘/’) characters to the alignment file^70^. The 3D-structures were checked and validated using SAVES v4 online tool (http://services.mbi.ucla.edu/SAVES/), root mean square deviations (RMSDs) respecting to the C-alpha trace of the template, and Discrete Optimized Protein Energy DOPE^71^. Finally, the three models obtained were minimized by AMBER v14 force field using the parameter settings by default (http://www.charmm-gui.org/).

### Functional divergence analysis

Protein-coding genes are modified by random mutations, which may then be affected by natural selection. The ‘covarion’-type models, also known as Type I functional divergence, detect differential rates (acceleration or deceleration) in each amino acid position^72^. The software DIVERGE 3.0^73^ was used to detect the functional divergence among different subgenotypes and genotypes of CSFV. The coefficients of Type-I functional divergence (θI) were calculated. If θI is significantly supported, it means that site-specific altered selective constraints or a radical shift of amino acid physiochemical property occurred after the diversification of the lineage. Moreover, a site-specific posterior analysis was used to predict amino acid residues that were crucial for functional divergence. In this analysis, large posterior probability (Qk) indicates a high possibility that the functional constraint (or the evolutionary rate) and/or the radical change in the amino acid property of a site is different between two clusters^73^. Since Type-II functional divergence describes a burst of rapid evolution immediately after gene duplication^74^ and this event has not been described in CSFV so far, we focused our analysis on Type I.

### Co-phylogenetic analysis

The presence of cophylogeny, the degree of co-speciation between Pestivirus species and their vertebrate hosts, was tested using A Novel Procrustes Application to Cophylogenetic Analysis (PACo)^75^. This novel tool produces a Procrustes superimposition plot enabling a graphical assessment of the fit of the parasite phylogeny onto the host phylogeny and a goodness-of-fit statistic, whose significance is established by randomization of the host-parasite association data. Three input files were provided, two of them contained the host and parasite phylogenies respectively, and the third one consisted of a binary matrix coding the host-parasite associations, the R script was run as described in the PACo Manual in Balbuena *et al*.^75^ available for free at http://www.uv.es/cophylpaco/index.html. In addition, a tree-reconciliation using Jane software v4^76^ was evaluated. Jane software assigns costs to four evolutionary events: cospeciation, duplication, host switch, and sorting. Additionally, a cost to failure to divergence is assigned^76^. Thus, the default cost settings were used.

## Electronic supplementary material

Supplementary Meterial

File S1
