# Supplementary material for: Deciphering the emergence, genetic diversity and evolution of classical swine fever virus
Source: Sci Rep. 2017 Dec 20;7:17887. doi: 10.1038/s41598-017-18196-y (PMC5738429; doi:10.1038/s41598-017-18196-y)
Supplement: Supplementary file 1 — Supplementary Meterial [file 41598_2017_18196_MOESM1_ESM.pdf]

Deciphering the emergence, genetic diversity and evolution of classical swine fever virus.

Liliam Rios[1¶], Liani Coronado[2¶], Dany Naranjo-Feliciano[2], Orlando Martínez-Pérez[3], Carmen L. Perera[2], Lilian Hernandez-Alvarez[2], Heidy Díaz de Arce[4], José I. Núñez[5], Llilianne Ganges[5], Lester J. Pérez[6]*

[1]*University of New Brunswick, Saint John, New Brunswick, E2L4L5, Canada.*

[2]*Centro Nacional de Sanidad Agropecuaria (CENSA), La Habana, 32700, Cuba*

[3]*Universidad de las Ciencias Informáticas (UCI), La Habana, Cuba*

[4]*Hospital Italiano de Buenos Aires, Juan D. Perón 4190, C1181ACH Buenos Aires, Argentina*

[5]*IRTA-CReSA. Centre de Recerca en Sanitat Animal, Barcelona, 08193, Spain*

[6]*Dalhousie University, Dalhousie Medicine New Brunswick, Saint John, New Brunswick, E2L4L5, Canada.*

[¶]These authors contributed equally to this work.

[*]Corresponding autor: Tel.:+1 (506)-636-6977 fax:+1 (506)-636-6258

E-mail address: lester.perez@dal.ca (Pérez, L.J.)

Supplementary Figures.

Fig. S1. Procrustean superimposition plot of Pestivirus members and their host. The ordinations of Pestivirus and their hosts are Principal Correspondence Coordinates of patristic distances. The Pestivirus configuration (dots) has been rotated and scaled to fit the hosts ordination (arrow tips). Length of arrows represents the projection of residuals onto the first two axes.

Fig. S2. Reconciliation of the Pestivirus phylogeny with that of their vertebrate hosts. Co-phylogenetic method implemented in Jane. The figure illustrates all possible codivergence, extinction, host-jumping events and lineage duplication events. Events: cospeciation is marked by a hollow colored circle; duplication is marked by a solid colored circle; host switch (host-jumping) is marked by a duplication, with an arrow following the trajectory of the switching species; failure to diverge is marked by a jagged line and a loss is marked by a dashed line.

FigureS1.

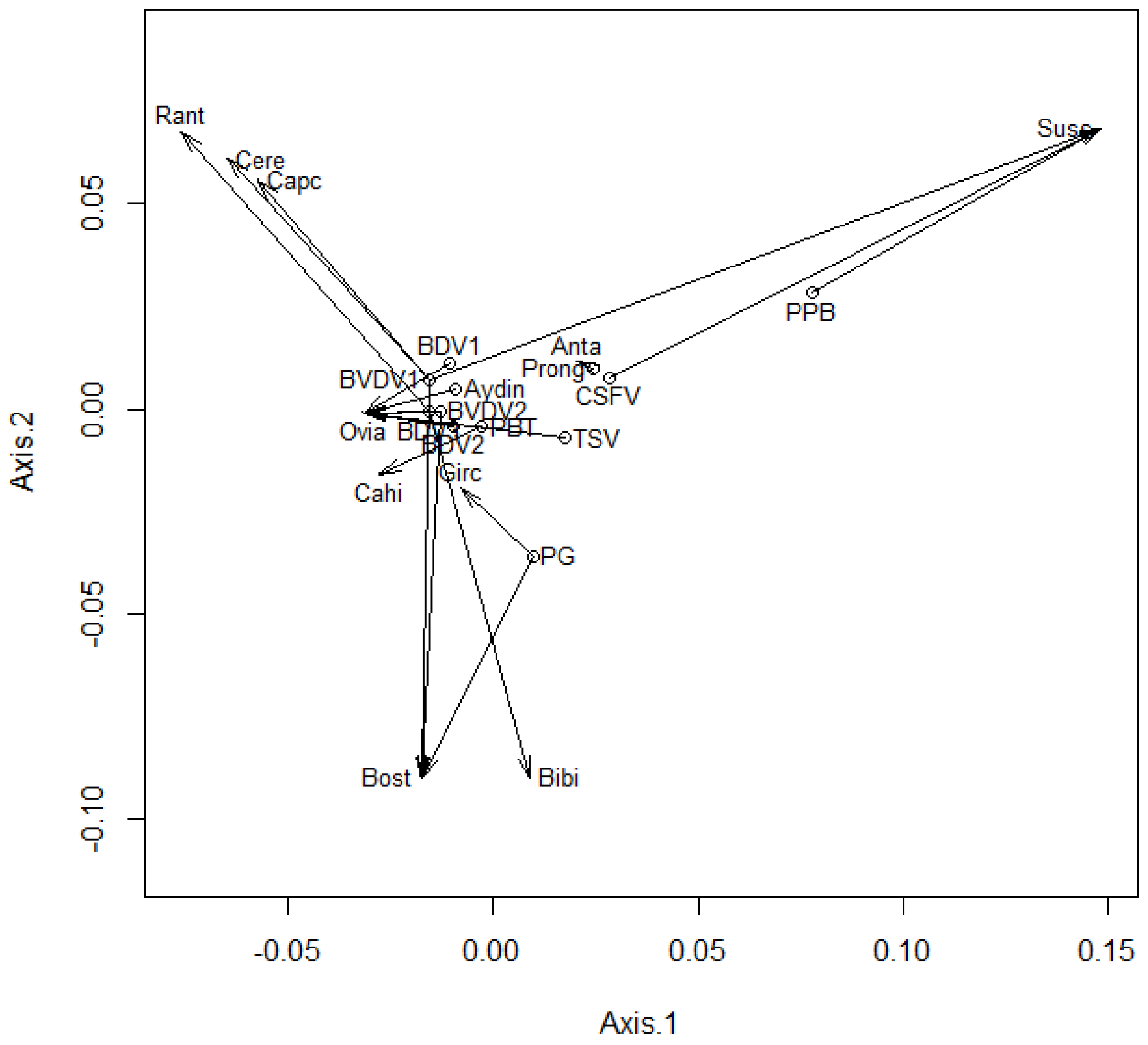
Rant
Cere
Capc
Susc
PPB
BDV1
Anta
Prong
CSFV
BVDV1
Aydin
BVDV2
Ovia
BDV2
TSV
Cahi
Girc
PG
Bost
Bibi
0.05
0.00
-0.05
-0.10
-0.05
0.00
0.05
0.10
0.15
Axis.1
Axis.2

FigureS2.

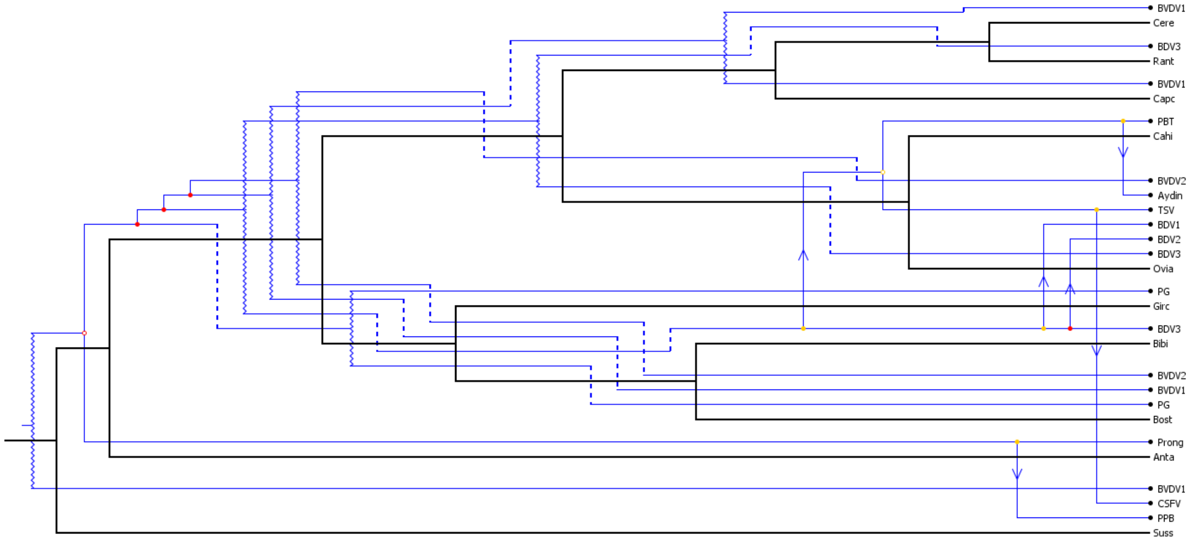
BVDV1
Cere
BDV3
Rant
BVDV1
Capc
PBT
Cahi
BVDV2
Aydin
TSV
BDV1
BDV2
BDV3
Ovia
PG
Girc
BDV3
Bibi
BVDV2
BVDV1
PG
Bost
Prong
Anta
BVDV1
CSFV
PPB
Suss

Table S1. Topology comparison for the different phylogenetic trees obtained from the molecular marker assessed using NJ, ML and BI methods.

Tree	Li	pKH	pSH	pRELL
1	*-117881.27*	*0*	*0*	*0*
2	*-104912.431*	*0*	*0*	*0*
3	*-100806.748*	*0*	*0*	*0*
4	*-92984.291*	*0*	*0*	*0*
5	*-91679.243*	*0*	*0*	*0*
6	*-91898.017*	*0*	*0*	*0*
7	-88583.945	0	0.208	0
8	-88390.306	0	0.338	0
9	-88283.454	0	0.442	0
10	-88122.586	0.061	0.723	0.061
11	**** -88020.792***	***-1***	***-1***	***0.939***
12	-88485.86	0	0.267	0
13	-88544.733	0	0.236	0
14	-89147.539	0	0.048	0
15	-88535.413	0	0.242	0

Li: log-likelihoods
pKH: P value for KH normal test (Kishino & Hasegawa 1989).
pRELL: RELL bootstrap proportions (Kishino & Hasegawa 1989)
pSH: P value with multiple-comparison correction (MC in table 1 of Shimodaira & Hasegawa 1999)
(-1 for P values means N/A)

TableS2. P-distance between Genotypes (Standart Error).

	I	II	III
**I**			
**II**	0.164 (0.009)		
**III**	0.153 (0.009)	0.175 (0.009)	

I, II and III: Indicates the CSFV genotypes 1,2 and 3 respectively.

TableS3. P-distance between subgenotypes (Standart Error).

	I.1	I.2	I.3	I.4	II.1	II.2	II.3
I.1							
I.2	0.080 (0.006)						
I.3	0.100 (0.008)	0.102 (0.008)					
I.4	0.107 (0.008)	0.105 (0.007)	0.115 (0.008)				
II.1							
II.2					0.101 (0.007)		
II.3					0.122 (0.008)	0.101 (0.007)	

I, II and III: Indicates the CSFV genotypes 1,2 and 3 respectively.

Table S4. Coalescent priors and clock models compared by log marginal likelihood.

**Clock Model**	**Coalescent**	**Path Sampling (PS)**	**Steping Stone (SS)**
SC	Const	-2515.67	-2515.79
SC	Exp	-2515.27	-2515.21
SC	Log	-2517.89	-2517.91
SC	BSP	-2518.16	-2517.89
UCDE	Const	-2515.69	-2515.58
**UCDE**	**Exp**	**-2514.42**	**-2514.53**
UCDE	Log	-2518.44	-2518.41
UCDE	BSP	-2517.88	-2517.76
UCDL	Const	-2515.82	-2515.86
UCDL	Exp	-2515.30	-2515.33
UCDL	Log	-2518.17	-2518.10
UCDL	BSP	-2515.91	-2515.86

The best model was highlighted in boldface. All comparisons were based on equal numbers of independent Monte Carlo samples. SC=strict clock, UCDL=uncorrelated log-normal, UCDE=uncorrelated exponential. Const=constant population size, Exp=exponentially growing population size, Log=Logistic growing population size, BSP=Bayesian skyline plot.

Table S5. Estimated substitution rates and time to the most recent common ancestor (tMRCA) for the CSFV strains.

Dataset of CSFV	Rate (s/s/y) (HPD95%)	tMRCA (y) (HPD95%)
Whole population	$1.33x10^{-3}(7.88x10^{-4}-1.98x10^{-3})$	1800 (1767-1896)
CSFV-G1	$2.66x10^{-4}(7.98x10^{-5}-5.02x10^{-4})$	1869 (1792-1915)
CSFV-G2	$6.37x10^{-4}(4.53x10^{-4}-8.45x10^{-4})$	1907 (1810-1942)
CSFV-G3	$1.31x10^{-2}(8.78x10^{-3}-1.92x10^{-2})$	1955 (1883-1973)

TableS6. Positively selected sites and parameters estimated by the CODEML program implemented in the PAML package.

**Gene**	**Model**	**lnL**	**Parameters**	**2LnL**	**Positive site**
E2	M1 (Neutral)	-12996.43561	p0= 0.87873 p1=0.12127		-
	M2 (Selection)	-12991.46514	ω= 20.97916 p0=0.81045 p1=0.12128 p2=0.00000	M2 vs M1 9.94ψψ	20*, 49*, 72**, 200**, 268*
	M7 (beta)	-12933.28143	p= 0.44371 q= 2.43618		-
	M8 (beta & w)	-12927.04105	ω= 7.07584 p0= 0.96075 p= 0.55985 q= 4.09560 (p1= 0.03925)	M8 vs M7 12.48ψψ	20*, 49*, 72**, 200**, 268*

*, codons with a posterior probability greater than 0.95 belonging to the positively selected class ($\omega > 1$); **, codons at which $P > 0.99$.
l, log-likelihood score
ψ: $P < 0.05$ ($\chi^2_{0.05,2} = 5.99$)
ψψ:$P < 0.01$ ($\chi^2_{0.01,2} = 9.21$)

TableS7. PAML branch-site model A analysis to identify branches under episodic positive selection in CSFV phylogenetic tree.

**Foreground branches**	**Parameters$^{n.m}$**	**-lnL$^{n.m}$**	**Parameters$^{a.m}$**	**-lnL$^{a.m}$**	**-2ΔlnL**	**Positively selected sites**
Subgenotype 1.1	P_0= 0.867<br>P_1= 0.120<br>P_{2a}= 0.011<br>P_{2b}= 0.001<br>ω_o= 0.079<br>ω_1= 1.000<br>ω_2=1.000	12996.046	P_0= 0.873<br>P_1= 0.119<br>P_{2a}= 0.007<br>P_{2b}= 0.001<br>ω_o= 0.079<br>ω_1=1.000<br>ω_2=**1.976**	12995.917	0.258	96, 168, 313
Subgenotype 1.2	P_0= 0.821<br>P_1= 0.114<br>P_{2a}= 0.057<br>P_{2b}= 0.008<br>ω_o= 0.071<br>ω_1= 1.000<br>ω_2= 1.000	12980.931	P_0= 0.873<br>P_1= 0.119<br>P_{2a}= 0.007<br>P_{2b}= 0.001<br>ω_o= 0.071<br>ω_1= 1.000<br>ω_2= 1.000	12981.060	0.258	4, 44, 49, 109, 157, 189, 193, 194, 201, 284, 366
Subgenotype 1.3	P_0= 0.879<br>P_1= 0.121<br>P_{2a}= 0.000<br>P_{2b}= 0.000<br>ω_o= 0.080<br>ω_1= 1.000<br>ω_2= 1.000	12996.825	P_0= 0.873<br>P_1= 0.119<br>P_{2a}= 0.007<br>P_{2b}= 0.001<br>ω_o= 0.080<br>ω_1= 1.000<br>ω_2= 1.000	12996.954	0.258	None
Subgenotype 1.4	P_0= 0.873<br>P_1= 0.119<br>P_{2a}= 0.007<br>P_{2b}= 0.001<br>ω_o= 0.079<br>ω_1= 1.000<br>ω_2= 1.000	12995.120	P_0= 0.873<br>P_1= 0.119<br>P_{2a}= 0.007<br>P_{2b}= 0.001<br>ω_o= 0.079<br>ω_1=1.000<br>ω_2= **7.471**	12992.085	**6.070****	**72, 175**
Subgenotype 2.1	P_0= 0.847<br>P_1= 0.116<br>P_{2a}= 0.032<br>P_{2b}= 0.004<br>ω_o= 0.079<br>ω_1= 1.000<br>ω_2= 1.000	12995.019	P_0= 0.873<br>P_1= 0.119<br>P_{2a}= 0.007<br>P_{2b}= 0.001<br>ω_o= 0.079<br>ω_1= 1.000<br>ω_2= 1.000	12995.149	0.26	248
Subgenotype 2.2	P_0= 0.762<br>P_1= 0.104<br>P_{2a}= 0.117<br>P_{2b}= 0.016<br>ω_o= 0.076<br>ω_1= 1.000<br>ω_2= 1.000	12991.230	P_0= 0.873<br>P_1= 0.119<br>P_{2a}= 0.007<br>P_{2b}= 0.001<br>ω_o=0.076<br>ω_1= 1.000<br>ω_2= 1.000	12986.543	**9.37****	**18, 22, 97, 100, 106, 119, 122, 124, 133, 146, 183, 206, 211, 240, 270, 272**
Subgenotype 2.3	P_0= 0.617<br>P_1= 0.084<br>P_{2a}= 0.264<br>P_{2b}= 0.036<br>ω_o= 0.073<br>ω_1= 1.000<br>ω_2= 1.000	12971.142	P_0= 0.873<br>P_1= 0.119<br>P_{2a}= 0.007<br>P_{2b}= 0.001<br>ω_o= 0.073<br>ω_1= 1.000<br>ω_2= 1.000	12966.455	**9.37****	**13, 27, 34, 36, 41, 58, 61, 72, 78, 107, 138, 141, 143, 147, 170, 184, 206, 214, 249, 250, 251, 266**

lnL: log-likelihood scores;

n.m: null model; a.m: alternative model

*$p<0.05$, χ^2= 3.84; **$p<0.01$, χ^2= 5.99

TableS7. PAML branch-site model A analysis to identify branches under episodic positive selection in CSFV phylogenetic tree.

Foreground branches	Parameters[n.m]	-lnL[n.m]	Parameters[a.m]	lnL[a.m]	-2ΔlnL	Positively selected sites
Subgenotype 3.1	P_0= 0.691<br>P_1= 0.094<br>P_{2a}= 0.189<br>P_{2b}= 0.026<br>ω_o= 0.078<br>ω_1= 1.000<br>ω_2= 1.000	12992.654	P_0= 0.873<br>P_1= 0.119<br>P_{2a}= 0.007<br>P_{2b}= 0.001<br>ω_o= 0.078<br>ω_1= 1.000<br>ω_2= 1.000	12992.654	0	None
Subgenotype 3.2	P_0= 0.801<br>P_1= 0.108<br>P_{2a}= 0.080<br>P_{2b}= 0.011<br>ω_o= 0.079<br>ω_1= 1.000<br>ω_2= 1.000	12997.731	P_0= 0.873<br>P_1= 0.119<br>P_{2a}= 0.007<br>P_{2b}= 0.001<br>ω_o= 0.079<br>ω_1= 1.000<br>ω_2=1.000	12997.731	0	None
Subgenotype 3.3	P_0= 0.873<br>P_1= 0.120<br>P_{2a}= 0.006<br>P_{2b}= 0.001<br>ω_o= 0.080<br>ω_1= 1.000<br>ω_2= 1.000	12996.424	P_0= 0.873<br>P_1= 0.119<br>P_{2a}= 0.007<br>P_{2b}= 0.001<br>ω_o= 0.080<br>ω_1= 1.000<br>ω_2= 1.000	12996.424	0	None

lnL: log-likelihood scores;
n.m: null model; a.m: alternative model
*$p<0.05$, χ^2= 3.84; **$p<0.01$, χ^2= 5.99

TableS8. Maximum Likelihood estimates of the coefficient of Type-I functional divergence (θ) from pairwise comparison among E2 CSFV-genotypes.

**Subgenotype**	**θML**	**θSE**	**θLTR**	**Qk**	**P**
Subgenotype 1.1/Subgenotype 1.2	0.9992	0.350583	0.984496	None	
**Subgenotype 1.1/Subgenotype 1.4**	**1.001116**	**0.127528**	**5.154724**	**113, 171**	**P<0.05**
Subgenotype 1.1/Subgenotype 2.1	0.9992	0.4283	1.136988	None	
Subgenotype 1.1/Subgenotype 2.2	0.9992	0.229132	1.698058	None	
**Subgenotype 1.1/Subgenotype 2.3**	**0.9992**	**0.126984**	**4.602288**	**113, 171**	**P<0.05**
Subgenotype 1.1/Subgenotype 3	0.224	0.126532	0.422494	None	
Subgenotype 1.2/Subgenotype 2.1	0.0528	0.780039	0.005261	None	
Subgenotype 1.2/Subgenotype 2.2	0.144	0.411028	0.13432	None	
**Subgenotype 1.2/Subgenotype 2.3**	**0.8408**	**0.233198**	**14.344478**	**36, 312**	**P<0.01**
**Subgenotype 1.2/Subgenotype 3**	**0.8744**	**0.234751**	**14.20064**	**174, 253**	**P<0.01**
**Subgenotype 1.4/Subgenotype 2.1**	**0.7192**	**0.285472**	**6.658923**	**78**	**P<0.01**
**Subgenotype 1.4/Subgenotype 2.2**	**0.5328**	**0.152342**	**5.102394**	**78**	**P<0.05**
**Subgenotype 1.4/Subgenotype 2.3**	**0.992**	**0.084824**	**23.718792**	**20, 36, 72, 74, 78, 192, 195, 268, 340**	**P<0.01**
**Subgenotype 1.4/Subgenotype 3**	**0.936716**	**0.085376**	**22.659187**	**74, 78, 174**	**P<0.01**
Subgenotype 2.1/Subgenotype 2.2	0.001	0.499921	0	None	
**Subgenotype 2.1/Subgenotype 2.3**	**0.8608**	**0.284609**	**12.039298**	**312**	**P<0.01**
**Subgenotype 2.1/Subgenotype 3**	**0.688**	**0.285138**	**7.970717**	**174**	**P<0.01**
**Subgenotype 2.2/Subgenotype 2.3**	**0.7264**	**0.149653**	**9.103527**	**36, 166**	**P<0.01**
**Subgenotype 2.2/Subgenotype 3**	**0.6016**	**0.151347**	**8.945452**	**174**	**P<0.01**
**Subgenotype 2.3/Subgenotype 3**	**0.832643**	**0.084961**	**25.007332**	**36, 174, 253, 312**	**P<0.01**

TableS9. Complete genome sequences of CSFV used in the current study.

GenBank	Strain	Country	isolation date	Size (nt)
X87939	Alfort/187	JOURNAL (Switzerland)	Submited (1995)	12298
U90951	Alfort A19	JOURNAL (France)	Submited (1997)	12298
EU490425	Thiverval	France	Submited (2008)	12321
EU789580|DQ415938|DQ656351	LOM	Japan	1980	12298
EU915211	flc-LOM	JOURNAL (Republic of Korea)	Submited (2008)	12298
D49533	GPE-	JOURNAL (Japan)	Submited (1995)	12298
AF326963	Eystrup	JOURNAL (Switzerland)	Submited (2000)	12301
CQ867021	Unknow	JOURNAL (France)	Submited (2004)	12301
NC_002657	Eystrup	JOURNAL (Switzerland)	Submited (2000)	12301
HM237795	CSFV/1.1/dp/CSF0382/XXXX/Koslov	Czech Republic	Submited (2010)	12298
D49532	ALD	JOURNAL (Japan)	Submited (1995)	12298
AY775178	Shimen/HVRI	China	Submited (2004)	12297
EU497410	JL1(06)	China	2006	12298
HQ380231	CSFV-GZ-2009	China	2009	12298
AF333000	cF114	JOURNAL (China)	Submited (2001)	12297
AF092448|AF121103|AF157635	Shimen	JOURNAL (China)	Submited (1999)	12298
DQ127910	SWH	China	Submited (2005)	12296
U45478	Glentorf	JOURNAL (Germany)	Submited (1996)	12278
X96550	CAP	JOURNAL (Switzerland)	Submited (1996)	12297
AF091661	Brescia	JOURNAL (Switzerland)	Submited (1998)	12297
U45477	Riems, C	JOURNAL (Germany)	Submited (1996)	12298
AY259122	Riems	JOURNAL (Switzerland)	Submited (2003)	12289
Z46258	Chinese strain (C-strain, EP 0 351 901 B1)	JOURNAL (The Netherlands)	Submited (1994)	12311
A47690	Unknow	JOURNAL (NL)	Submited (1995)	12311
HM175885	C-ZJ-2008	China	2008	12311
AY382481	Chinese vaccine strain	JOURNAL (China)	Submited (2003)	12310
AF531433	HCLV	JOURNAL (China)	Submited (2002)	12310
AY805221	C/HVRI	China	Submited (2004)	12310
AY663656	Unknow	Unknow	Submited (2004)	12310
EU857642	HCLV	India	Submited (2008)	12311
AF352565	LPC	JOURNAL (Taiwan)	Submited (2001)	12344
AY578687	BRESCIAX	Brescia Plum Island, USA	2001	12285
M31768	Brescia	JOURNAL(The Netherlands)	Submited (1990)	12283
AF099102|AF132116	CS	JOURNAL (Rusia)	Submited (2000)	12310
AY578688	RUCSFPLUM	CS Plum Island, USA	2001	12308
AY646427	94.4/IL/94/TWN	Taiwan	1994	12296
L49347	P97	Unknow	Unknow	12144
GU324242	CSFV/2.3/wb/XXX0609/2004/Uelzen	Germany	2004	12297

JQ595295	-	Belgium	1993-1994	12262
HQ148062	-	Bulgaria	2007	12296
AX191158	ALFORT	JOURNAL (NL)	Submited (2001)	12284
A77196	ALFORT	JOURNAL (NL)	Submited (1994)	12284
J04358	Alfort/Tuebingen	Germany	1999	12297
A16790	Unknow	JOURNAL (NL)	Submited (1990)	12284
GU233733	-	Germany	2009	12297
GU233734	-	Germany	2009	12297
GU233732	-	Germany	2005	12297
GU233731	-	Germany	2006	12297
FJ265020	-	Spain	2001	12299
HQ148061	CSFV/2.3/dp/CSF0821/2002/HR/Novska	Croatia	2002	12295
GQ902941	Paderborn	JOURNAL (Denmark)	Submited (2009)	12296
AY072924	Paderborn	JOURNAL (Denmark)	Submited (2002)	12229
AY554397	96TD	Taiwan	Submited (2004)	12296
GQ923951	SXCDK	China	2009	12296
KC149991	-	South Korea	2011	12295
FJ529205	Zj0801	China	2008	12296
JQ268754	-	China	2010	12296
KC149990	-	South Korea	2011	12296
GU592790	HEBZ	China	2009	12296
AY568569	0406/CH/01/TWN	Taiwan	Submited (2004)	12296
GQ122383	SXYL2006	China	2006	12295
AY367767	GXWZ02	China	Submited (2003)	12296
HQ148063	-	Lithuania	2009	12296
JX262391	-	China	2011	12296
JX218094	-	China	2012	12296

TableS10. CSFV sequence of the E2 complete gene used in the current study

GenBank	Strain	Country	isolation date
AJ704817	Margarita	Cuba	1993
JX028201	Margarita	Cuba	1993
JX028202	CSF1056 Holguin	Cuba	2009
JX028203	CSF1057 Santiago de Cuba	Cuba	2011
JX028204	CSF1058 Pinar del Rio	Cuba	2010
AF134209	BKK/88	Thailand	1988
JQ411570	CSF0306	Malaysia	1986
AF134207	BKK/50	Thailand	1950
AF134208	BKK/91	Thailand	1991
AY578688	RUCSFPLUM	CS Plum Island	2001
M31768	Brescia	Italy	1945
AY578687	BRESCIAX	Italy	2001
JQ411588	CSF0947 Brescia	Italy	1951
HQ380240	CSFV-NH-2009	China	2009
HQ380241	CSFV-PY-2009	China	2009
DQ127910	SWH/CA/2004	China	2004
HQ380231	CSFV-GZ-2009	China	2009
EU497410	JL1(06)	China	2006
HQ380234	CSFV-DL-2009	China	2009
AY775178	Shimen/HVRI	China	1945
HQ380233	CSFV-XT-2010	China	2010
HQ380232	CSFV-DB-2009	China	2009
HQ380239	CSFV-GZh-2009	China	2009
HQ380236	CSFV-PR-2008	China	2008
HQ380237	CSFV-DaB-2008	China	2008
HQ380235	CSFV-DH-2008	China	2008
HQ380238	CSFV-FS-2009	China	2009
HQ380242	CSFV-SS-2010	China	2010
FJ598612	Shimen-ZJ	China	2008
X71780	Weybridge	GB	1954
AF134210	KPP/93	Thailand	1993
KF007902	SH111	India: Rani, Assam	2012
HQ380243	CSFV-JY-2010	China	2010
X87939	Alfort/187	France	1968
EU789580|DQ415938|DQ656351	LOM	Japan	1980
GQ396706	TW/05	Taiwan	2005
AF326963	Eystrup	Germany	1964
U45478	Glentorf	Germany	1968
U45477	Riems, C vaccine	Germany	1984
AY663656	CWH	China	2003
FJ598611	C-strain-ZJ	China	2008
HM175885	C-ZJ-2008	China	2008
JQ595295	Wingene	Belgium	1993-1994
JQ411564	CSF0104 Diepholz I	Germany	1994
HQ148061	CSFV/2.3/dp/CSF0821/2002/HR/Novska	Croatia	2002
JQ411559	CSF0002 Atzbuell	Germany	1984
JQ411572	CSF0372	Czech Republic	1996
JQ411569	CSF0291	Poland	1995
JQ411560	CSF0014	Germany	1989
DQ907717	LN1.84	China	1984
JQ411562	CSF0073	Austria	1990
JQ411561	CSF0021	Germany	1989

JQ411567	CSF0283	Netherlands	1997
JQ411566	CSF0277	Germany	1997
GQ923951	SXCDK	China	2009
AY526727	83-s106	Taiwan	1994
HQ697222	GDDG.2008	China	2008
JQ001833	HNHY11	China	2011
JQ411582	CSF0708	United Kingdom	2000
JQ411591	CSF1048 Panevezys	Lithuania	2009
HQ148063	CSFV/2.1/dp/CSF1048/2009/LT/Panevezys	Lithuania: Panevezys	2009
GQ122383	SXYL2006	China	2006
HQ697224	GDFS.2009	China	2009
HQ697225	GDGZ.2009	China	2009
FJ456873	JX2-06	China	2006
EF683616	JX-05	China	2005
DQ907716	ZJ10.2005	China	2005
EF683608	HZ2-04	China	2004
FJ456874	SX1-06	China	2006
EF683609	HZ2-06	China	2006
AY568569	0406/CH/01/TWN	Taiwan	2004
FJ607779	ZS1-08	China	2008
FJ607780	ZS2-08	China	2008
FJ582643	HZ2-08	China	2008
FJ582644	FY-08	China	2008
FJ529205	Zj0801	China	2008
FJ582642	HZ1-08	China	2008
FJ598609	QZ1-08	China	2008
FJ598610	QZ2-08	China	2008
EF683621	SH2-05	China	2005
DQ907714	ZJ7.2005	China	2005
DQ907713	ZJ6.2005	China	2005
DQ907715	ZJ8.2005	China	2005
EF683623	SX-04	China	2004
EF683606	HuZ2-05	China	2005
KC149991	PC11WB	South Korea	2011
JQ268754	Heb52010	China	2010
KC149990	YC11WB	South Korea	2011
EF683611	JH-05	China	2005
GU592790	HEBZ	China	2009
FJ456872	SX2-06	China	2006
EF683617	LS-05	China	2005
EF683613	JX1-06	China	2006
FJ456871	HZ-05	China	2005
FJ456868	JX-07	China	2007
FJ456870	JX-04	China	2004
FJ456867	HZ1-06	China	2006
EF683618	QZ1-06	China	2006
FJ456866	HZ1-07	China	2007
EF683620	SH1-07	China	2007
FJ456865	SH2-07	China	2007
EF683612	JS-07	China	2007
EF683615	JX4-06	China	2006
EF683622	SX3-06	China	2006
EF683610	HZ2-07	China	2007
FJ977628	JX1-09	China	2009
EF683619	QZ2-06	China	2006

JQ411571	CSF0309 Kanagawa	Japan	1974
L49347	p97/FL/94/TWN	Taiwan	1994
AY646427	94.4/IL/94/TWN	Taiwan	1994
AY526731	79-60	Taiwan	1990
AY526730	85-12A	Taiwan	1996
JQ411575	CSF0410 Congenital Tremor	United Kingdom	1964

TableS11. Sequence of the E2 complete gene for the different Pestivirus

Pestivirus	GenBank ID	Year of collection
Border disease virus	NC_003679	1987
Pestivirus sp. Bison-1	AF144619	1996
Border disease virus 1	AY163656	1992
Border disease virus 1	AY163655	1994
Border disease virus 3	AY163660	1999
Border disease virus 2	AY163659	1999
Border disease virus 2	AY163658	2000
Border disease virus 2	AY163657	1985
Border disease virus	KC484999	2007
Tunisian sheep virus	AY452482	2000
Porcine pestivirus isolate Bungowannah	NC_023176	2003
Pestivirus Burdur/05-TR	KM408491	2005
Bovine viral diarrhea virus type 1	NC_001461	1988
Bovine viral diarrhea virus type 2	LC006970	1991
Bovine viral diarrhea virus type 1	AF144616	1991
Bovine viral diarrhea virus type 1	AF144615	1986
Bovine viral diarrhea virus type 1	AF144614	1980
Bovine viral diarrhea virus type 1	AF144611	1981
Bovine viral diarrhea virus type 1	AF144610	1993
Bovine viral diarrhea virus type 1	AF144609	1996
Bovine viral diarrhea virus type 2	AF144613	1993
Bovine viral diarrhea virus type 2	AF144612	1997
Bovine viral diarrhea virus type 1	AF526381	1995
Bovine viral diarrhea virus type 2	AF145967	1993
Bovine viral diarrhea virus type 1	M96687	1967
Pestivirus Giraffe-1	NC_003678	1967
Pestivirus reindeer-1	AF144618	1996
Pestivirus Giraffe-1	KJ660072	1990
Pronghorn antelope pestivirus	NC_024018	2003
Bovine viral diarrhea virus type 2	HG426495	2007
Bovine viral diarrhea virus type 2	HG426494	2010
Bovine viral diarrhea virus type 2	HG426493	2010
Bovine viral diarrhea virus type 2	HG426492	2010
Bovine viral diarrhea virus type 2	HG426491	2000
Bovine viral diarrhea virus type 2	HG426487	2013
Bovine viral diarrhea virus type 2	HG426485	2013
Bovine viral diarrhea virus type 2	HG426483	2013
Bovine viral diarrhea virus type 2	HG426481	2013
Bovine viral diarrhea virus type 2	HG426479	2013
Pestivirus strain Aydin	JX428945	2004
Classical swine fever virus	AJ704817	1993
Classical swine fever virus	JX028201	1993
Classical swine fever virus	JX028202	2009
Classical swine fever virus	JX028204	2010
Classical swine fever virus	JX028203	2011
Classical swine fever virus	U45478	1968
Classical swine fever virus	AY578687	2001
Classical swine fever virus	AF326963	1964
Classical swine fever virus	X71780	1954
Classical swine fever virus	M31768	1945
Classical swine fever virus	X87939	1968
Classical swine fever virus	JQ411588	1951
Classical swine fever virus	HQ380231	2009

Classical swine fever virus	EU497410	2006
Classical swine fever virus	AY775178	1945
Classical swine fever virus	EU789580	1980
Classical swine fever virus	DQ127910	2004
Classical swine fever virus	U45477	1984
Classical swine fever virus	JQ411570	1986
Classical swine fever virus	AY663656	2003
Classical swine fever virus	KF007902	2012
Classical swine fever virus	GQ396706	2005
Classical swine fever virus	AF134208	1991
Classical swine fever virus	HQ380243	2010
Classical swine fever virus	JQ411575	1964
Classical swine fever virus	HQ380242	2010
Classical swine fever virus	HQ380238	2009
Classical swine fever virus	HQ380235	2008
Classical swine fever virus	HQ380239	2009
Classical swine fever virus	HQ380233	2010
Classical swine fever virus	HQ380232	2009
Classical swine fever virus	HQ380241	2009
Classical swine fever virus	AF134207	1950
Classical swine fever virus	JQ411566	1997
Classical swine fever virus	HQ380237	2008
Classical swine fever virus	HQ380236	2008
Classical swine fever virus	FJ598612	2008
Classical swine fever virus	JQ411567	1997
Classical swine fever virus	JQ411561	1989
Classical swine fever virus	AF134210	1993
Classical swine fever virus	HQ380240	2009
Classical swine fever virus	HQ380234	2009
Classical swine fever virus	HM175885	2008
Classical swine fever virus	AY646427	1994
Classical swine fever virus	AF134209	1988
Classical swine fever virus	JQ411571	1974
Classical swine fever virus	L49347	1994
Classical swine fever virus	JQ411591	2009
Classical swine fever virus	JQ411572	1996
Classical swine fever virus	JQ411559	1984
Classical swine fever virus	GQ923951	2009
Classical swine fever virus	GQ122383	2006
Classical swine fever virus	HQ697224	2009
Classical swine fever virus	JQ411569	1995
Classical swine fever virus	AY568569	2004
Classical swine fever virus	JQ411582	2000
Classical swine fever virus	KC149991	2011
Classical swine fever virus	KC149990	2011
Classical swine fever virus	HQ148061	2002
Classical swine fever virus	GU592790	2009
Classical swine fever virus	JQ595295	1994
Classical swine fever virus	JQ411564	1994
Classical swine fever virus	HQ697222	2008
Classical swine fever virus	AY526727	1994
Classical swine fever virus	JQ268754	2010
Classical swine fever virus	FJ529205	2008
Classical swine fever virus	DQ907716	2005
Classical swine fever virus	HQ697225	2009

Classical swine fever virus	DQ907717	1984
Classical swine fever virus	DQ907714	2005
Classical swine fever virus	DQ907715	2005
Classical swine fever virus	DQ907713	2005
Classical swine fever virus	FJ456873	2005
Classical swine fever virus	FJ456874	2006
Classical swine fever virus	EF683609	2006
Classical swine fever virus	AY526731	1990
Classical swine fever virus	EF683608	2004
Classical swine fever virus	AY526730	1996
Classical swine fever virus	FJ456868	2007
Classical swine fever virus	FJ456865	2007
Classical swine fever virus	EF683616	2005
Classical swine fever virus	EF683615	2006
Classical swine fever virus	FJ456870	2004
Classical swine fever virus	EF683622	2006
Classical swine fever virus	EF683613	2006
Classical swine fever virus	FJ456866	2007
Classical swine fever virus	FJ977628	2009
Classical swine fever virus	FJ456872	2006
Classical swine fever virus	FJ456867	2006
Classical swine fever virus	EF683620	2007
Classical swine fever virus	EF683619	2006
Classical swine fever virus	EF683618	2006
Classical swine fever virus	EF683617	2005
Classical swine fever virus	EF683610	2007
Classical swine fever virus	EF683612	2007
Classical swine fever virus	EF683623	2004
Classical swine fever virus	EF683611	2005
Classical swine fever virus	FJ582642	2008
Classical swine fever virus	EF683606	2005
Classical swine fever virus	JQ001833	2011
Classical swine fever virus	FJ598609	2008
Classical swine fever virus	FJ456871	2005
Classical swine fever virus	FJ598610	2008
Classical swine fever virus	EF683621	2005
Classical swine fever virus	FJ607779	2008
Classical swine fever virus	FJ582643	2008
Classical swine fever virus	FJ607780	2008
Classical swine fever virus	FJ582644	2008
Classical swine fever virus	JQ411560	1989
Classical swine fever virus	JQ411562	1990

TableS12. Sequence of the cytochrome B of vertebrate hosts

**Pestivirus**	**GenBank ID**
Bison bison	AF036273.1
Bos taurus	D34635.1
Capra hircus	D84201.1
Capreolus capreolus	KJ681491.1
Cervus elaphus	JF489133.1
Antilocapra americana sonoriensis	GU175434.1
Giraffa camelopardalis	AY121992.1
Ovis aries	AB006800.1
Rangifer tarandus	DQ673135.1
Sus scrofa	AB015083.1
